# Sex- and age-specific sensitivities of the endocannabinoid system in Alzheimer's disease revealed by PET imaging with [^18^F]FMPEP-*d*_2_ and [^18^F]MAGL-2102

**DOI:** 10.7150/thno.106592

**Published:** 2025-02-18

**Authors:** Anna Pees, Christopher Daniel Morrone, Junchao Tong, Jian Rong, Tuo Shao, Darcy Wear, Steven H. Liang, Wai Haung Yu, Neil Vasdev

**Affiliations:** 1 Azrieli Centre for Neuro-Radiochemistry, Brain Health Imaging Centre, Campbell Family Mental Health Research Institute, Centre for Addiction and Mental Health (CAMH), Toronto, ON, Canada, M5T 1R8.; 2 Department of Radiology and Imaging Sciences, Emory University, Atlanta, Georgia, United States.; 3 Jiangsu Key Laboratory of Infection and Immunity, Suzhou Medical College, Suzhou, China; 4 Department of Pharmacology & Toxicology, University of Toronto, Ontario, Canada.; 5 Department of Psychiatry, University of Toronto, Ontario, Canada.; 6 Division of Nuclear Medicine and Molecular Imaging, Massachusetts General Hospital & Department of Radiology, Harvard Medical School, Boston, Massachusetts 02114, United States.

**Keywords:** endocannabinoid system, cannabinoid receptor 1, monoacylglycerol lipase, Alzheimer's disease, PET

## Abstract

The endocannabinoid system is a critical brain signaling pathway that is dysregulated in various brain disorders, including Alzheimer's disease (AD). Cannabinoid-targeted therapies and imaging approaches have gained increasing interest; however, the biological impact of the endocannabinoid system in disease needs further validation. We aimed to study changes in cannabinoid receptor 1 (CB1) and monoacylglycerol lipase (MAGL), components of endocannabinoid signaling and degradation, in a mouse model of AD by PET imaging.

**Methods:** [^18^F]FMPEP-*d*_2_ and [^18^F]MAGL-2102 were produced on a commercial radiosynthesis module. PET-CT images with both tracers were acquired in a knock-in mouse model of AD bearing mutated human amyloid precursor protein (*App^NL-G-F^*) at 3 ages, and compared to wild-type mice. Excised brains were used for *in vitro* autoradiography with [^18^F]FMPEP-*d*_2_ and [^18^F]MAGL-2102, immunofluorescence, and western blotting. Male wild-type and 5xFAD mice were chronically treated with MAGL inhibitor JZL184 and imaged with [^18^F]MAGL-2102 two days after ending treatment.

**Results:** PET imaging showed sex-, age- and genotype-dependent changes in CB1 and MAGL availability. At 4-months (early-stage β-amyloid pathology), female *App^NL-G-F^* mice had lower CB1 availability, and MAGL availability was increased in male *App^NL-G-F^*_,_ compared to wild-types_._ At 8-months, no genotype differences in CB1 were observed, yet MAGL availability was reduced in *App^NL-G-F^* frontal cortex, and male *App^NL-G-F^* mice exhibited higher MAGL than transgenic females brain-wide. At 12-months (late-stage β-amyloid pathology), significantly lower uptake of [^18^F]FMPEP-*d*_2_ was observed in *App^NL-G-F^* compared to wild-type, with no changes in [^18^F]MAGL-2102 binding. *App^NL-G-F^* plaque staging was confirmed by Thioflavin-S staining. Imaging findings were supplemented by autoradiography, immunofluorescence, and western blots. [^18^F]MAGL-2102 availability was responsive to target engagement of the MAGL inhibitor JZL184 in wild-type and 5xFAD mice.

**Conclusions:** The present study showed dynamic age-, sex- and pathology-related changes in CB1 and MAGL availability from early-stage β-amyloid pathology, suggesting that the endocannabinoid system is a useful target for diagnostics and treatment of AD. Finally, these results highlight that endocannabinoid sex differences should be considered in diagnostics and drug development.

## Introduction

The endocannabinoid system (ECS) is a lipid-based signaling system within the central nervous system which regulates an array of physiological and cognitive functions including neurodevelopment, neuronal plasticity, pain, inflammation, stress and emotion 1,2. It comprises two cannabinoid receptors, type-1 and type-2 (CB1 and CB2, respectively), their endogenous ligands 2-arachidonoylglycerol (2-AG) and anandamide (AEA), and enzymes involved in synthesis and degradation, such as diacylglycerol lipase (DAGL; 2-AG synthesis), monoacylglycerol lipase (MAGL; 2-AG degradation) and fatty acid amide hydrolase (FAAH; AEA degradation) 3. The ECS is also involved in several psychiatric and neurological disorders such as Alzheimer's disease (AD), addictions, schizophrenia and post-traumatic stress disorder, and has been investigated for novel therapeutic and diagnostic approaches 4,5. Recent therapeutic efforts focus on the modulation of ECS enzymes, allowing endocannabinoid levels to be influenced indirectly, with fewer side effects 4. To study changes in receptor and enzyme levels of the ECS *in vivo*, positron emission tomography (PET) radiopharmaceuticals targeting CB1, CB2, MAGL and FAAH have been translated for human studies 1,2,6.

There are conflicting data on CB1 and other ECS components in AD models and patients, but in general, CB1 levels decrease with disease (reviewed in 7,8). A PET imaging study in a rodent model of AD showed age- and genotype-dependent alterations in [^18^F]FMPEP-*d*_2_ binding to CB1 9, while a human study with [^18^F]MK-9470 and [^11^C]PIB found no correlation between CB1 availability and β-amyloid (Aβ) deposition 10. While changes in MAGL availability in AD have not been reported using PET, MAGL inhibition in AD mice improves cognitive function, decreases Aβ burden and reduces neuroinflammation 11. To better understand the ECS involvement in AD and support development of ECS-targeted therapies, this study aimed to investigate changes in CB1 and MAGL expression and availability in an aging transgenic mouse model of AD by PET imaging using [^18^F]FMPEP-*d*_2_ 12 and [^18^F]MAGL-2102, 13, respectively, and validate results with autoradiography (ARG), immunofluorescence (IF), and western blotting.

To model early through robust stages of Aβ plaque pathology, we utilized the *App^NL-G-F^* knock-in mouse model. *App^NL-G-F^* mice express human amyloid precursor protein with 3 familial AD mutations (Swedish, Iberian, Arctic) which increase Aβ production, aggregation and the Aβ42:40 ratio. Because the ECS is sexually dimorphic and strongly influenced by hormonal factors in rodents as well as humans, 14 and as AD is known to affect women more than men implying sex differences in underlying mechanisms 15, this study was powered for sex as a biological variable and its potential interaction with AD pathology.

## Methods

### Animals

Animal experiments were conducted in accordance with the ethical standards of the Canadian Council on Animal Care (CAMH, protocol #871) or the Institutional Animal Care and Use Committee (Massachusetts General Hospital, protocol #2020N000001). Mice were housed in a 12-h light:dark cycle with *ad libitum* access to chow and water. Transgenic *App^NL-G-F/NL-G-F^* knock-in mice (Riken institute 16) were bred in-house. Wild-type (wt) mice (C57BL/6) were purchased from Jackson Laboratory. Male and female mice were imaged at 4-, 8- or 12-months with [^18^F]FMPEP-*d*_2_ and [^18^F]MAGL-2102. Brains were excised and flash-frozen with one hemisphere sub-dissected. Fresh-frozen whole hemispheres were cryosectioned (Leica CM3050S; 10μm sagittal). All ordered mice were allowed to habituate to the facility for at least 2 weeks prior to data collection.

### PET imaging

Automated radiosyntheses of [^18^F]FMPEP-*d*_2_ 17 and [^18^F]MAGL-2102 (based on the manual radiosynthesis 13; detailed procedure in **Supplementary methods**) were conducted. Mice underwent PET imaging with [^18^F]MAGL-2102, followed by [^18^F]FMPEP-*d*_2_ (3-7 days between; **Tables 1-2** for experimental details and *n*). For each scan, mice were catheterized in the tail vein and positioned in a dedicated small animal PET/CT (computed tomography) scanner (nanoScan™, Mediso Ltd., Budapest, Hungary). Tracer formulations were quickly taken up in a syringe, diluted with saline to a concentration of about 5 MBq/200µL if needed, and injected. Anaesthesia was maintained throughout the scanning period while monitoring body temperature and respiration. CT was acquired before each PET scan and used for attenuation and scatter correction purposes as well as PET/CT co-registration and co-registration with a stereotactic MR atlas of mouse brain 18 to define anatomical regions of interest (ROI). Dynamic PET scans were acquired for 120 minutes immediately after i.v. administration of the respective tracer.

The acquired list mode data were sorted into 39 frames (3 × 5, 3 × 15, 3 × 20, 7 × 60, 17 × 180, and 6 × 600 s) 3D true sinograms (ring difference 84). The 3D sinograms were converted into 2D sinograms using Fourier-rebinning and reconstructed using a 2D-filtered back projection (FBP) with a Hann filter at a cut-off of 0.50 cm^-1^. Static images of the complete emission acquisition were reconstructed with the manufacturer's proprietary iterative 3D algorithm (six subsets and four iterations). All image data were corrected for detector geometry and efficiencies, dead-time and decay-corrected to the start of acquisition, with corrections for attenuation and scatter using a CT-based material map.

Image analyses and extraction of brain time-activity curves (TACs) from the dynamic filtered back projection (FBP) images were performed using PMOD (version 4.203, Zurich, Switzerland) and an MR-based mouse brain atlas 18. Standardized uptake values (SUV) were calculated by normalizing regional radioactivity to injected radioactivity and body weight. Analysis focused on caudate putamen, frontal cortex, parietal-temporal cortex, occipital cortex, medulla, midbrain, pons, thalamus, hippocampus, whole brain (minus cerebellar cortex), and cerebellar cortex. Averages of TACs from left and right hemisphere were used for further analyses.

### Autoradiography

Brain sections were pre-incubated with 50 mM Tris-HCl (pH = 7.6) (+0.01% Triton X-100 for [^18^F]MAGL-2102) for 10 min. Tissue sections were subsequently incubated with [^18^F]FMPEP-*d*_2_ (0.5-5 nM) in 50 mM Tris-HCl (pH = 7.6), or [^18^F]MAGL-2102 (0.17-4.6 nM) in 50 mM Tris-HCl (pH = 7.6) +0.01% Triton X-100, each with or without a 10 μM blocking agent (unlabelled tracer) for 90 min, followed by three washes in 50 mM Tris-HCl (pH = 7.6) for 1 min and a dip in ice-cold deionized water. Slides were air-dried and exposed to phosphor screens (BAS-IP TR4020; GE Healthcare) overnight. Screens were scanned with an Amersham Typhoon phosphorimager (GE Healthcare) and images were analyzed using MCID 7.0 imaging suite (Interfocus Imaging, Cambridge, UK). Sectioning artifacts were excluded during analysis.

### Immunofluorescence

Fresh-frozen 10 μm rodent sagittal brain sections (1 section per mouse containing cortex, hippocampus and striatum) were brought to room temperature and post-fixed in 4% paraformaldehyde for 20 min. After drying, sections were washed three times in 1× phosphate buffered saline (PBS; pH 7.4) for 10 min, followed by antigen retrieval in heated (85 °C) sodium citrate buffer (10 mM, pH 6) +0.05% tween. Sections were washed three times in 1×PBS for 10 min and incubated for 1 h with a blocking solution of 5% goat serum, 0.1% triton X-100 and 1% BSA in PBS. Next, the primary incubation was carried out overnight with either rabbit anti-CB1 (1:200; Abcam, ab23703) or rabbit anti-MAGL (1:100; Abcam, ab24701) in the blocking solution. Sections were washed three times in 1×PBS for 10 min, followed by the secondary incubation for 2 h with goat anti-rabbit 568 (1:200; Invitrogen, A11011) diluted in blocking solution. Sections were washed again three times in 1×PBS for 10 min before incubating for 7 min with 1% Thioflavin-S (Sigma-Aldrich, T1892) solution. After two washes with 70% ethanol for 5 min and three washes with PBS for 5 min, DAPI (1:5000 in PBS; Roche Diagnostics, 10236276001) was applied for 10 min. After three PBS washes for 5 min and a final rinse in distilled H_2_O, the sections were cover slipped using Prolong Gold anti-fade mounting media (Invitrogen, P36930). Antibody details are also provided in Table S1.

Slides were imaged using an Olympus VS200 slide scanner. Hippocampal CB1 and DAPI were analyzed in ImageJ. Images (20x) were normalized for brightness/contrast, converted to 8-bit, inverted, and underwent signal:noise thresholding and binarization. Pixel density was then quantified using the “analyze particles” function in ImageJ, in the total hippocampus, combined CA1 and CA3 pyramidal cell layers (PCL), and granular cell layer (GCL) to identify the area (µm^2^) of positive staining, which was normalized to hippocampal area (µm^2^) and expressed as a percentage.

### Western blot

Sub-dissected frontal cortex, hippocampus and striatum tissue were weighed and homogenized in 1xRIPA buffer (10 µl/mg) containing protease inhibitor (1:100; Sigma-Aldrich, P8340), phenylmethylsulfonyl fluoride (PMSF; 100mM stock added at 1:100; Sigma-Aldrich, P7626), sodium fluoride (NAF; 100mM stock added at 1:100; Sigma-Aldrich, S7920), sodium orthovanadate (Na_3_VO_4_; 100mM stock added at 1:100; Sigma-Aldrich, S6508) and EGTA (100mM stock added at 1:100; Sigma-Aldrich, E3889), by sonication at 30% duty cycle for 5 seconds, repeated x3. Brains were centrifuged at 5000g for 15 minutes at 4°C, aliquoted and stored at -80°C. Bicinchonic acid (BCA) assay (Pierce™ BCA Protein Assay Kits, ThermoFisher Scientific, 23227) was utilized to quantify protein concentrations. Samples were prepared with dithiothreitol (DTT) (ThermoFisher, Canada, R0861, 1M, 1:10), LDS 4x dye (ThermoFisher, Canada, NP0008, 1:4), and distilled water to load 10 μg protein then heated for 6 minutes at 70°C. Gel electrophoresis (125 volts) was run on 4-20% tris-glycine polyacrylamide gels (Invitrogen, WXP42026BOX) with Tris-Glycine SDS Running buffer (Novex^TM^, Invitrogen, LC2675), then protein was transferred to 0.2 µm nitrocellulose membranes in chilled transfer buffer (1x tris-glycine, 20% methanol) at 200 mA for 2 hours. Membranes were rinsed in ddH_2_O, blocked in 5% milk in TBS-T (tris-buffered saline with 0.05% Tween 20) for 1 hour at room temperature. Following 3 TBS-T washes, primary incubation was conducted overnight at 4°C: rabbit anti-CB1 (1:1000; Abcam, ab23703) and mouse anti-SNAP25 (1:1000; Abcam, ab66066), or rabbit anti-MAGL (1:100; Abcam, ab24701), diluted in superblock (Thermo Scientific, 37535). Membranes were washed, incubated with secondary antibodies conjugated to horseradish peroxidase: goat anti-rabbit IgG and/or goat anti-mouse IgG+IgM (both 1:10000; Jackson Immunoresearch Labs, 111-035-008 and 115-035-044, respectively) for 1 hour, washed again then incubated for 5 minutes in ECL (Cytiva, RPN2106). Following imaging, membranes were washed, stripped for 15 minutes at room temperature (Restore^TM^ PLUS, Thermo Scientific, 46430), washed, blocked in 5% milk in TBS-T, and re-probed for mouse anti-β-actin (1:5000; Invitrogen, AM4302), with subsequent steps performed as described above. Antibody details are also provided in Table S1.

Imaging was conducted on Amersham ImageQuant 800 (Cytiva) and densitometric analyses in ImageJ for CB1 (3 bands, 53-60 kDA), MAGL (2 bands, 33-35 kDA), SNAP25 (1 band, 25 kDA) which were normalized to β-actin (1 band, 42 kDA). The ImageJ densitometric analysis involved binarization of images and the generation of a signal peak per band, relative to the background signal of the membrane. Images of the full blots are included in **Figures S13** and **S14**.

### MAGL inhibition with JZL184

A separate cohort of 6-month-old male wt and 5xFAD mice (both ordered from Jackson Laboratory) was treated with JZL184 (Tocris Bioscience), a potent and selective MAGL inhibitor, or vehicle, and imaged with [^18^F]MAGL-2102. Specifically, adult mice (2-4 months old) were injected with JZL184 (12 mg/kg dissolved in saline, 10% Tween-80, 10% DMSO) or vehicle intraperitoneally 3x per week, until 6-months of age (*n* = 3/genotype/treatment, all male); adapted from a previous report on JZL184 11. Two days following the end of treatment, mice were imaged with [^18^F]MAGL-2102. For **Figure 6**, summed images were generated from % injected dose per cubic centimetre (0-60 min).

### Statistical analysis

Sample sizes were determined by the authors' past experiences with *in vivo* and tissue experiments. Two-way ANOVA with Holm-Šídák post-hoc tests were performed on the area under the curve (AUC) of the TACs to examine the difference in radiotracer uptake/washout between the different groups. Two-tailed unpaired t-tests were also performed on select AUC data to further support the findings. Three-way ANOVA was utilized to assess JZL184 treatment effect on [^18^F]MAGL-2102 SUV (0-60 min) in **Figure 6**.

IF data was analyzed with multiple unpaired t-tests with the Holm-Šídák correction for hippocampal CB1 and DAPI in total hippocampus, GCL and PCL. Western blot data was analyzed with two-tailed unpaired t-test for genotype effect when there was no sex effect. When sex or genotype*sex differences were present for the western data, statistical analysis was run with two-way ANOVA, with Holm-Šídák post-hoc when appropriate. Age, genotype and age*genotype western analysis was conducted on 4- and 12-month mice with two-way ANOVA (Holm-Šídák post-hoc). Pearson's correlations between CB1 and [^18^F]FMPEP-*d*_2_, and MAGL and [^18^F]MAGL-2102 PET data were conducted, with a linear regression (best fit line with 95% confidence intervals) when significant. Four data points (2 from CB1 and SNAP-25 gels; 2 from MAGL gels) were excluded from western analysis due to technical errors. Significant differences were *P < 0.05, **P < 0.01; and trending if #P < 0.10.

## Results

[^18^F]FMPEP-*d*_2_ 12 was synthesized according to a simplified procedure reported by our lab 17, which entailed an automated two-step one-pot synthesis using ditosylmethane-*d*_2_ and phenoxy precursor on a GE Tracerlab FX N synthesis module. [^18^F]FMPEP-*d*_2_ was obtained with a radiochemical yield of 8±1% (decay-corrected, n = 8), a molar activity (A_m_) of 322±101 GBq/µmol and radiochemical purity >95% within 70 minutes. [^18^F]MAGL-2102 was obtained in a copper-mediated reaction of [^18^F]fluoride with a boronic ester precursor according to the recently reported manual synthesis 13, with minor modifications (see **Supplementary methods**) including automation on a commercial (GE Tracerlab FX 2N) synthesis module. [^18^F]MAGL-2102 was obtained with radiochemical yield of 14±4% (dc, n = 4), A_m_ of 506±302 GBq/µmol and radiochemical purity >95%.

*App^NL-G-F^* mice 16 were scanned at 3 stages of Aβ pathology [4-month (early), 8-month (mid) and 12-month (late)] with [^18^F]FMPEP-*d*_2_ and [^18^F]MAGL-2102, compared to wild-type controls. These ages were selected for Aβ plaque density in this model as determined by Thioflavin-S staining (**Figure S1**). Regions of interest focused on the frontal cortex, hippocampus and caudate putamen (striatum), which have high expressions of CB1 and MAGL and are affected by Aβ plaques in AD 19. Additionally, the parietal-temporal cortex, occipital cortex, medulla, midbrain, pons, thalamus, cerebellum, and the whole brain were analyzed.

### [^18^F]FMPEP-*d*_2_ imaging reveals reduced CB1 at early-stage Alzheimer's pathology

**Figure 1:**
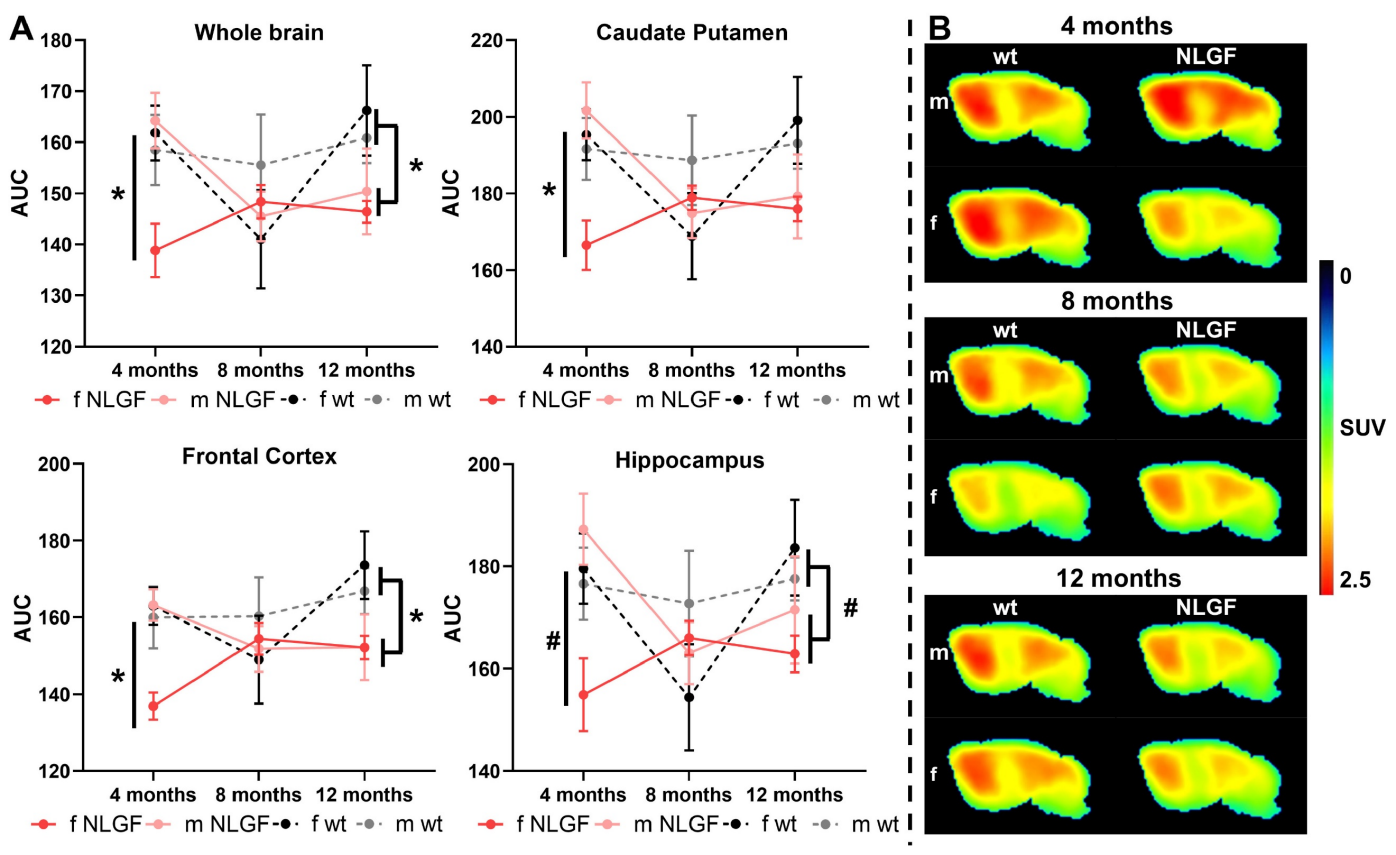
** A)** AUCs of [^18^F]FMPEP-*d*_2_ TACs for *App^NL-G-F^* and wt mice (m/f) at 4-, 8- and 12-months, for whole brain, caudate putamen, frontal cortex and hippocampus. Mean±SEM, *P < 0.05 and #P < 0.10 for genotype comparisons. **B)** Average summed (0-120 min) images of [^18^F]FMPEP-*d*_2_ in brain of wt and *App^NL-G-F^* mice of both sexes (m/f) at 4-, 8- and 12-months of age. Shown are sagittal images at the right side of brain (2 mm from middle line). **Abbreviations:** AUC: area under the curve; m/f: male, female; NLGF: *App^NL-G-F^*; SUV: standardized uptake value; TAC: time activity curve; wt: wild-type.

[^18^F]FMPEP-*d*_2_ exhibited good brain uptake (>1 SUV), with the highest in the caudate putamen and the lowest in medulla and cerebellum (**Figure 1, Figure S2**), and is consistent with previous reports in rodents 9. At 4-months, a significant genotype*sex interaction was detected in the whole brain (***P = 0.0282***, F(1,12) = 6.226), with reduced CB1 availability in female *App^NL-G-F^* compared to male *App^NL-G-F^* mice (***P = 0.0176***), and compared to female wt mice (***P = 0.0304***). This interaction effect was consistent in the caudate putamen (***P = 0.0187***, F(1,12) = 7.378), frontal cortex (***P = 0.0197***, F(1,12) = 7.224) and hippocampus (***P = 0.0269***, F(1,12) = 6.354; **Figure 1A**), and was significant or trending in most other brain regions (**Table 3**). At 8-months, no significant differences in CB1 availability were detected by sex or genotype, with trends to a genotype*sex interaction effect in the midbrain (*P = 0.0831*, F(1,13) = 3.523), thalamus (*P = 0.0973*, F(1,13) = 3.192) and cerebellum (*P = 0.0841*, F(1,13) = 3.498; **Figure 1A, Table S2**). These trends are potentially due to lower CB1 availability in 8-month female wt mice, which was also notable in comparison to their 4- and 12-month counterparts. At 12-months, a significant genotype effect was observed in the frontal cortex (***P = 0.0290***; F(1,13) = 6.022), medulla (***P = 0.0362***, F(1,13) = 5.456) and pons (***P = 0.0323***, F(1,13) = 5.740), demonstrating reduced CB1 availability in male and female *App^NL-G-F^* compared to wt mice. This effect was trending across the whole brain (*P = 0.0523*, F(1,13) = 4.563), and in the midbrain (*P = 0.0680*, F(1,13) = 3.962), thalamus (*P = 0.0592*, F(1,13) = 4.273) and cerebellum (*P = 0.0944*, F(1,13) = 3.256), but was non-significant in the caudate putamen (P = 0.1615, F(1,13) = 2.204), hippocampus (P = 0.1141, F(1,13) = 2.870), and other brain regions (**Figure 1A; Table 4**). With statistical grouping of 12-month male and female mice (no sex differences were observed at 12-months, see **Table 4**), we detected a trend in the hippocampus (unpaired t-test: *P = 0.0902*, t = 1.811, df = 15) and a significant loss in 12-month *App^NL-G-F^* mice across the whole brain (unpaired t-test: ***P = 0.0358***, t = 2.307, df = 15). Average summed [^18^F]FMPEP-*d*_2_ images demonstrate the loss of CB1 signal in 4-month female *App^NL-G-F^* mice, no genotype change at 8-months, and reduced availability in *App^NL-G-F^* mice of both sexes at the 12-month late-stage AD pathology (**Figure 1B**). Complete [^18^F]FMPEP-*d*_2_ two-way ANOVA statistics for each age and region are reported in **Table 3** (4-month), **Table S2** (8-month), and **Table 4** (12-month).

### [^18^F]MAGL-2102 imaging reveals increased MAGL at early-stage pathology

**Figure 2:**
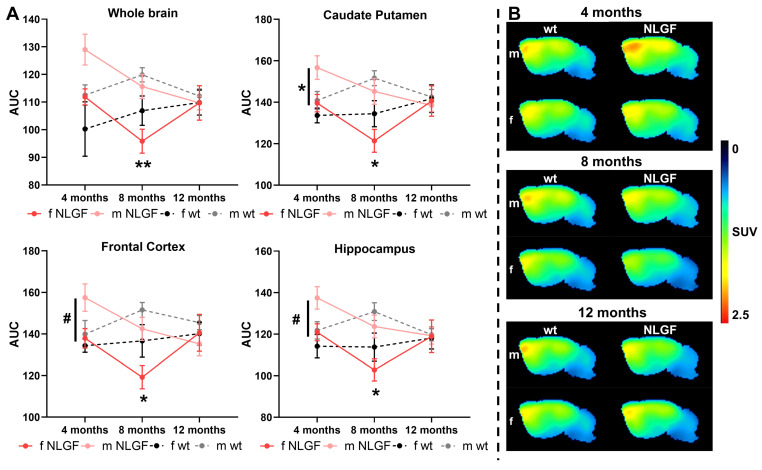
**A)** AUCs of [^18^F]MAGL-2102 TACs for *App^NL-G-F^* and wt mice (m/f) at 4-, 8- and 12-months, for whole brain, caudate putamen, frontal cortex and hippocampus. Mean±SEM, #P < 0.10, *P < 0.05 for *App^NL-G-F^* sex comparisons. **B)** Average summed (0-120 min) images of [^18^F]MAGL-2102 in brain of wt and *App^NL-G-F^* mice of both sexes (m/f) at 4-, 8- and 12-months of age. Shown are sagittal images at the right side of brain (2 mm from middle line).** Abbreviations:** AUC: area under the curve; m/f: male, female; NLGF: *App^NL-G-F^*; SUV: standardized uptake value; TAC: time activity curve; wt: wild-type.

[^18^F]MAGL-2102 showed the highest uptake in the frontal cortex and caudate putamen (**Figure 2, Figure S3**), consistent with previous reports in rodents and non-human primates 13. Brain regions with the lowest uptake were the medulla, pons and cerebellum. Notably, early-stage male *App^NL-G-F^* mice exhibit an increased [^18^F]MAGL-2102 availability. Significant whole brain sex (***P = 0.0337***, F(1,12) = 5.749) and genotype (***P = 0.0405***, F(1,12) = 5.270) effects were detected at 4-months, with similar significant differences seen in the caudate putamen (sex: ***P = 0.0192***, F(1,12) = 7.311; genotype: ***P = 0.0323***, F(1,12) = 5.860), frontal cortex (sex: ***P = 0.0406***, F(1,12) = 5.264; genotype: *P = 0.0760*, F(1,12) = 3.772), hippocampus (sex: ***P = 0.0292***, F(1,12) = 6.128; genotype: ***P = 0.0405***, F(1,12) = 5.272), thalamus (sex: ***P = 0.0162***, F(1,12) = 7.802; genotype: ***P = 0.0317***, F(1,12) = 5.907) and consistent trends in all other brain regions except the medulla and pons (**Table 5**). Male *App^NL-G-F^* mice demonstrate regional specific increased MAGL availability compared to female *App^NL-G-F^* mice (caudate putamen: ***P = 0.0390***; frontal cortex: *P = 0.0519*, hippocampus*: P = 0.0660*; thalamus ***P = 0.0382***) and to male wt mice (caudate putamen: *P = 0.0562*; hippocampus: *P = 0.0828*; thalamus: *P = 0.0608*; **Figure 2A, Table 5**).

*App^NL-G-F^* MAGL levels decrease between 4- and 8-months. Furthermore, significant sex effects were found across the whole brain at 8-months (***P = 0.0026***, F(1,18) = 12.22) mainly from significantly lower MAGL availability in female *App^NL-G-F^* compared to male *App^NL-G-F^* mice (***P = 0.0051***); the sex effects were found consistently across all separate brain regions (**Table 6**). The wt sex comparison was non-significant in the whole brain (P = 0.1892), but trending in the midbrain (*P = 0.0824*) and thalamus (*P = 0.0890*). A significant genotype effect was detected in the frontal cortex only at 8-months (***P = 0.0464***, F(1,18) = 4.577), with lower MAGL in *App^NL-G-F^* mice of both sexes compared to wt mice (**Figure 2A, Table 6**). Finally, at 12-months no significant sex or genotype differences in MAGL availability were detected in any region, with a trend to reduced cerebellar availability in females (*P = 0.0862,* F(1,12) = 3.495; **Figure 2A, Table S3**). Average summed [^18^F]MAGL-2102 images demonstrate the notable increase in MAGL availability in 4-month male *App^NL-G-F^* mice compared to female transgenics and wt mice, which then decreases at 8- and 12-months (**Figure 2B**). Complete [^18^F]MAGL-2102 two-way ANOVA statistics for each age and region are reported in in **Table 5** (4-month), **Table 6** (8-month), and **Table S3** (12-month).

### [^18^F]FMPEP-*d*_2_ and [^18^F]MAGL-2102 autoradiography

**Figure 3:**
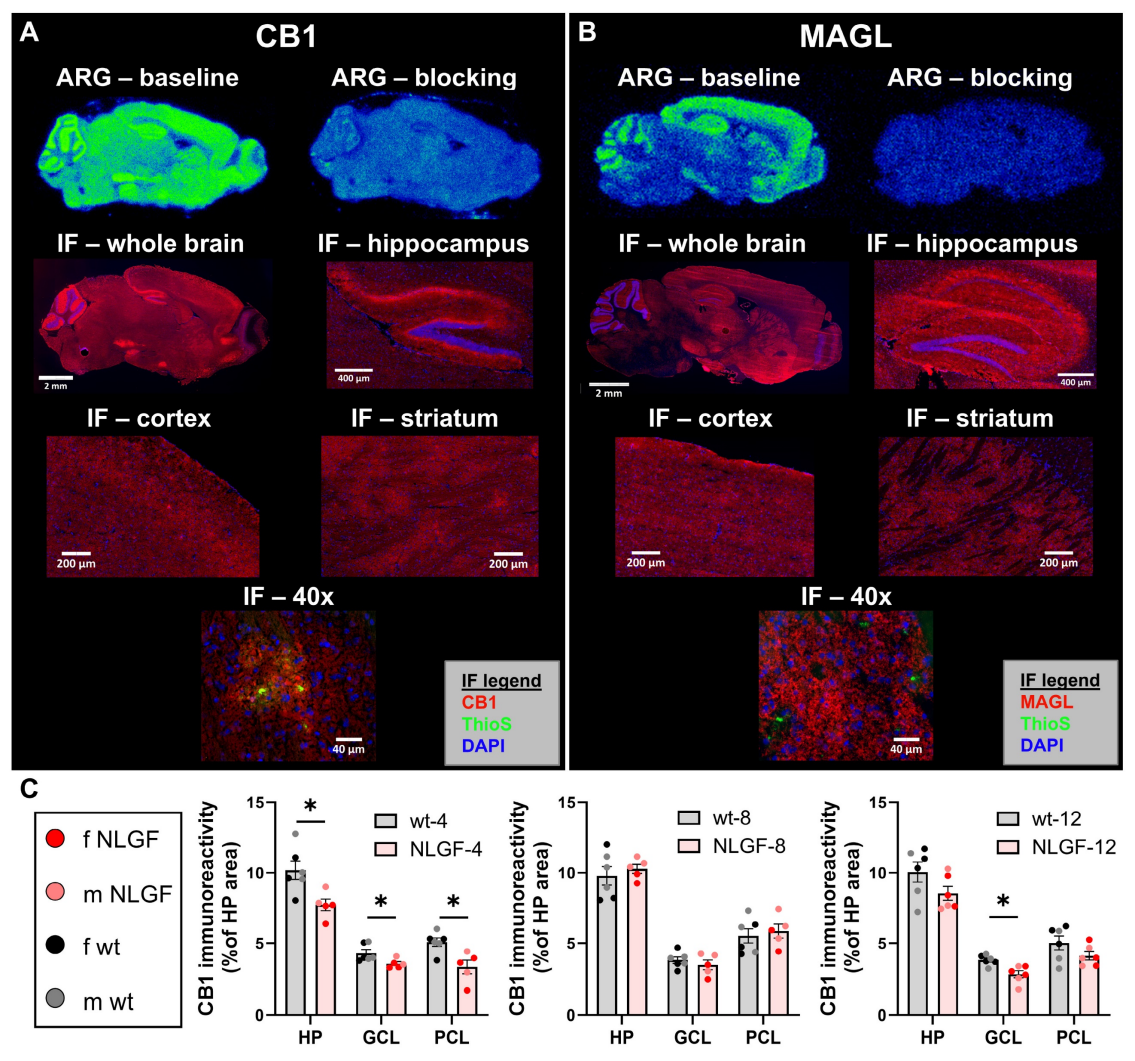
Representative images of ARG (baseline and blocking with cold self) with [^18^F]FMPEP-*d*_2_ (**A**) and [^18^F]MAGL-2102 (**B**) in sagittal brain sections of 12-month *App^NL-G-F^* mice. IF in sequential sections for CB1 (**A;** red, left) and MAGL (**B;** red, right) at 10x (whole brain), 20x (hippocampus, cortex, caudate putamen) and striatal 40x images demonstrating CB1, but not MAGL, specificity around plaques (Thioflavin-S, green). **C)** Hippocampal CB1 IF quantification reveals a loss in *App^NL-G-F^* mice at 4-months, no change at 8-months, and a loss again at 12-months (mean +/- SEM; *n* = 5-6/genotype/age). All images in panel **A** are from the same female 12-month* App^NL-G-F^* mouse. All images in panel **B** are from the same male 12-month* App^NL-G-F^* mouse. **Abbreviations:** ARG: autoradiography; CB1: cannabinoid receptor 1; GCL: granular cell layer; HP: hippocampus; IF: immunofluorescence; m/f: male, female; MAGL: monoacylglycerol lipase; NLGF: *App^NL-G-F^*; PCL: pyramidal cell layer; ThioS: Thioflavin-S; wt: wild-type.

To confirm tracer specificity and the suitability of ARG assay conditions, a baseline-blocking experiment was first performed on one section per group (12/condition). [^18^F]FMPEP-*d*_2_ showed an average of 45% (36-54%) blocking after pre-incubation with 10 µM unlabelled FMPEP-*d*_2_ (**Figure 3, Figure S4**). Tracer binding in the ARG study was found across all brain regions, and is consistent with PET imaging data. [^18^F]MAGL-2102 showed on average 55% (39-68%) blocking (**Figure 3, Figure S5**) when sections were pre-incubated with 10 µM unlabelled MAGL-2102 and is also consistent with literature findings (37-70%, 13). Baseline ARG distribution matched the literature 13, and uptake patterns in [^18^F]MAGL-2102 PET, with high binding in cerebral cortices, striatum, hippocampus, thalamus and cerebellar cortex. The relatively high non-specific binding of both tracers can be attributed to the high lipophilicity, in particular of [^18^F]FMPEP-*d*_2_, which was previously determined (logD_7.4_(MAGL) = 3.7, 13 logD_7.4_(FMPEP-*d*_2_) = 4.2) 20.

An additional ARG study was carried out to detect inter-group differences. Sections (*n* = 3/sex/genotype/age) were incubated with [^18^F]FMPEP-*d*_2_ (**Figure S4**) or [^18^F]MAGL-2102 (**Figure S5**) and binding was assessed by signal intensity in whole brain, cortex and hippocampus. We found high variation in signal intensity within groups (coefficient of variation range: [^18^F]FMPEP-*d*_2_ whole brain: 3-44%, cortex: 2-50%, hippocampus 4-44%; [^18^F]MAGL-2102 whole brain: 7-40%, cortex: 3-41%, hippocampus 13-43%), which may be attributed to variable non-specific binding observed in blocking experiments as well as to variability in drawing regions of interest of the brain regions, and no significant inter-group differences. For [^18^F]MAGL-2102 we were able to observe trends in cortex and whole brain that support the PET imaging data (**Figure S6**), especially regarding sex differences.

### ECS protein levels reflect age-related compensation and impairments in *App^NL-G-F^* mice

CB1 and MAGL immunostaining aligned regionally (e.g. cortex, hippocampus, striatum) with areas of high uptake in ARG (**Figure 3A,B**). CB1 showed a ubiquitous distribution across the whole brain section, with higher signal in dystrophic neurites surrounding plaques (40x image) and in hippocampal regions with known high CB1 density 21: intense signal was observed in processes/dendrites synapsing on excitatory neurons of granular and pyramidal cell layers (GCL; PCL), and diffusely through the hippocampus. MAGL showed widespread distribution over the whole brain section with high signal intensity in the hippocampus, cerebral cortices, striatum and thalamus, and did not show specificity around plaques (40x image).

CB1 immunoreactivity was analyzed in the hippocampus: 4-month *App^NL-G-F^* mice exhibit reduced CB1 within the total hippocampus, GCL and PCL (***P = 0.0298***, ***P = 0.0382***, ***P = 0.0298***, respectively). Conversely, at 8-months, hippocampal CB1 is unchanged by genotype (all P = 0.8111), and the *App^NL-G-F^* mice are increased compared to 4-month early-stage pathology. The 12-month mice exhibit no genotype differences in total hippocampal or the PCL CB1 (both P = 0.2087), and a significant loss of CB1 in the GCL (***P = 0.0188***; **Figure 3C**). Consistent with PET imaging, this demonstrates the sensitivity of CB1 to Aβ pathology in *App^NL-G-F^* mice, with a loss of hippocampal CB1 from early-stage plaque pathology (4-months) and preceding DAPI+ PCL loss which does not occur until the late-stage (see **Figure S7**).

**Figure 4:**
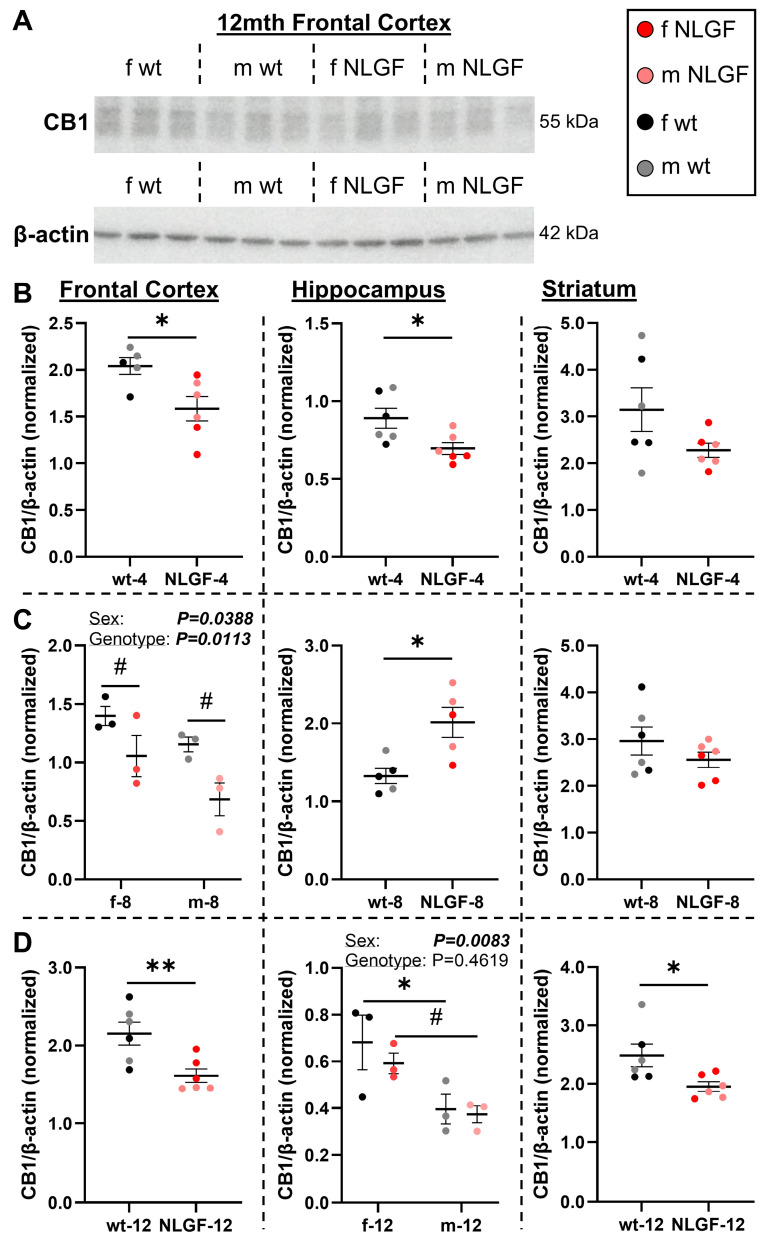
**A)** Representative blot of CB1 and β-actin. **B)** In 4-month *App^NL-G-F^* mice, CB1 protein levels are significantly reduced in the frontal cortex and hippocampus, and trending down in the striatum. **C)** At 8-months, *App^NL-G-F^* mice exhibit reduced frontal cortex CB1, compensation in the hippocampus, and no change in the striatum. **D)** At 12-months, CB1 is significantly reduced in the *App^NL-G-F^* frontal cortex and striatum, but not the hippocampus. Sex differences were detected in the 8-month frontal cortex and 12-month hippocampus, with less CB1 protein in males in both cases. Normalized protein values are relative to exposure time and should not be compared between gels and graphs. Within each region, 4- and 12-month mice were on the same gels; see **Figure S12** for age comparisons. Mean+/-SEM (*n* = 5-6/genotype/age).** Abbreviations:** CB1: cannabinoid receptor 1; kDa: kilodalton; m/f: male, female; NLGF: *App^NL-G-F^*; wt: wild-type.

**Figure 5:**
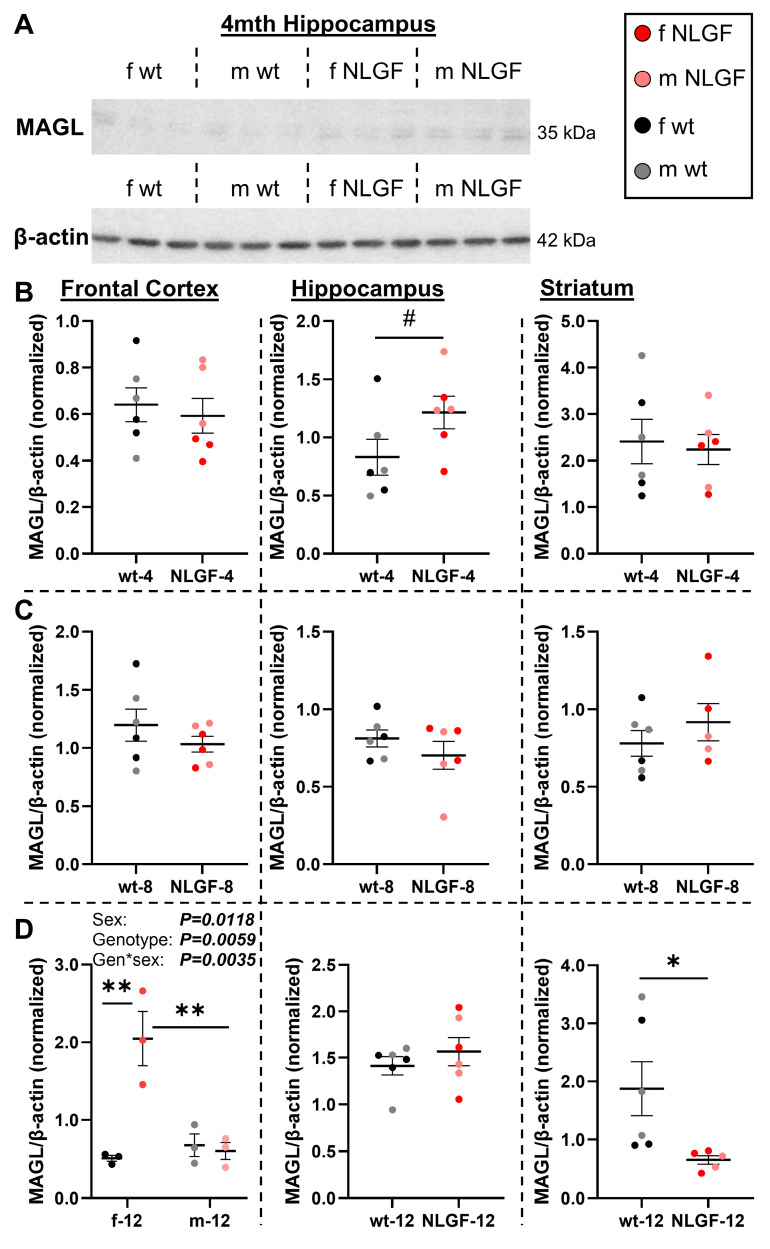
**A)** Representative blot of MAGL and β-actin. **B)** 4-month *App^NL-G-F^* mice exhibit no differences in frontal cortical or striatal MAGL protein levels, with a trend to higher hippocampal MAGL levels. **C)** No genotype differences were detected at 8-months in any region. **D)** At 12- months, female *App^NL-G-F^* mice exhibit increased MAGL protein levels. In the hippocampus, no changes were observed at 12-months. Striatal MAGL is reduced in 12-month *App^NL-G-F^* mice. Normalized protein values are relative to exposure time and should not be compared between gels and graphs. Within each region, 4- and 12-month mice were on the same gels; see **Figure S12** for age comparisons. Mean+/-SEM (*n* = 5-6/genotype/age).** Abbreviations:** gen: genotype; kDa: kilodalton; m/f: male, female; MAGL: monoacylglycerol lipase; NLGF: *App^NL-G-F^*; wt: wild-type.

PET was next aligned to CB1 and MAGL protein levels with western blotting (**Figures 4-5**). We detected a CB1 reduction in the *App^NL-G-F^* frontal cortex compared to wt (**Figure 4A**). At 4-months, CB1 protein was significantly lower in the frontal cortex (***P = 0.0226***), hippocampus (***P = 0.0257***), and trending to a reduction in the striatum (P = 0.1077; **Figure 4B**). Genotype (***P = 0.0113***, F(1,8) = 10.72) and sex differences were detected in the 8-month frontal cortex (***P = 0.0388***, F(1,8) = 6.093) with lower CB1 in* App^NL-G-F^* mice and in males. In the 8-month hippocampus, *App^NL-G-F^* mice exhibit increased CB1 (***P = 0.0127***) supportive of compensatory increases seen in hippocampal CB1 staining. No differences were detected in the 8-month striatum (P = 0.2726; **Figure 4C**). At 12-months, *App^NL-G-F^* mice have reduced CB1 protein in the frontal cortex (***P = 0.0099***), no genotype changes in the hippocampus (P = 0.4619, F(1,8) = 0.5971), and significantly lower striatal CB1 (***P = 0.0294***). A significant sex effect was detected in the 12-month hippocampus (***P = 0.0083***, F(1,8) = 12.14), with lower levels in male wt mice compared to females (***P = 0.0460***) and trending lower in male vs. female transgenics (*P = 0.0659*; **Figure 4D**). Quantification of the presynaptic protein SNAP-25 demonstrated that CB1 reductions were primarily related to a specific loss within the cannabinoid pathway, and not necessarily from a generalized loss of presynaptic protein/synapses (**Figure S8**). In summary, these data indicate that *App^NL-G-F^* mice exhibit a loss of CB1 protein levels in the frontal cortex from early-stage pathology, a loss followed by a compensatory response in the hippocampus, and a reduction in the striatum starting at late-stage pathology.

MAGL protein levels were assessed (**Figure 5A**). Frontal cortical MAGL levels were unchanged between wt and *App^NL-G-F^* mice at 4-months (P = 0.6536). In the hippocampus, a trend to increased MAGL was observed in the grouped 4-month *App^NL-G-F^* mice (*P = 0.0949*; **Figure 5B**), which was significantly higher when comparing just the males (*n* = 3/genotype, unpaired t-test, ***P = 0.0425***). The representative 4-month hippocampal blot demonstrates the trend to higher MAGL levels in *App^NL-G-F^* mice, especially in males (**Figure 5A**), supportive of the [^18^F]MAGL-2102 results in early-stage pathology (**Figure 2**). MAGL protein levels in the striatum were unchanged by the *App^NL-G-F^* genotype at 4-months (P = 0.7682; **Figure 5B**). No MAGL protein differences were detected at 8-months in frontal cortex (P = 0.3117), hippocampus (P = 0.3219), or striatum (P = 0.3597; **Figure 5C**). 12-month genotype (***P = 0.0059***, F(1,8) = 13.78), sex (***P = 0.0118***, F(1,8) = 10.51) and genotype*sex interaction (***P = 0.0035***, F(1,8) = 16.72) effects were detected in the frontal cortex, with increased MAGL in female *App^NL-G-F^* compared to female wt (***P = 0.0011***) and compared to male *App^NL-G-F^* mice (***P = 0.0017***). In the 12-month hippocampus, no differences in MAGL were detected by genotype (P = 0.4114), with a significant striatal loss in 12-month *App^NL-G-F^* compared to controls (***P = 0.0418*; Figure 5D**).

CB1 and MAGL protein levels were analyzed for potential correlations with [^18^F]FMPEP-*d*_2_ and [^18^F]MAGL-2102 availabilities (**Figure S9**). Correlations were grouped by samples which ran together on a gel: by age (4- and 12-month grouped, 8-month separate) and region (frontal cortex, hippocampus, and caudate putamen (striatum) separately). For CB1 protein with [^18^F]FMPEP-*d*_2_, a significant positive correlation was detected in the frontal cortex of the grouped 4- and 12-month mice (***P = 0.0324***, r^2^ = 0.2190). No other correlations were detected for [^18^F]FMPEP-*d*_2_, and none for MAGL protein with [^18^F]MAGL-2102 (see **Figure S9** for plots). The ratio of radiotracer availability to protein expressed was calculated for each age and region for [^18^F]FMPEP-*d*_2_:CB1 (**Figure S10**) and [^18^F]MAGL-2102:MAGL (**Figure S11**). For CB1, trends to a higher imaged availability per protein level in early- (frontal cortex and hippocampus) and mid-stages (frontal cortex) was observed compared to wt mice (**Figure S10**). These analyses support the notion that an early *App^NL-G-F^* deficit promotes a compensatory response with trafficking of protein reserves to active sites. However, when the CB1 protein levels were higher than wt in the 8-month hippocampus (**Figure 4C**), the availability:protein instead trended down in the AD mice, an early sign that this compensation and reserve was less effective, or approaching exhaustion (**Figure S10**). For *App^NL-G-F^* MAGL, significantly lower availability per expression was detected in the 8-month striatum, yet this was increased in the 12-month striatum, compared to wt mice. In tandem with the age and genotype trends (**Figure 2** and **5**), this suggests [^18^F]MAGL-2102 availability changes as an earlier sign of MAGL loss than protein levels.

The effects of age were also assessed for CB1, MAGL and SNAP-25 protein levels, comparing 4- and 12-month-old mice. These data highlight an age-associated decline in hippocampal CB1 protein, higher wild-type hippocampal MAGL with age (trending in *App^NL-G-F^*), and reduced striatal MAGL protein levels in the late-stage *App^NL-G-F^* mice; see **Figure S12** for complete analyses and statistics.

### Therapeutic MAGL inhibition measured by PET

**Figure 6:**
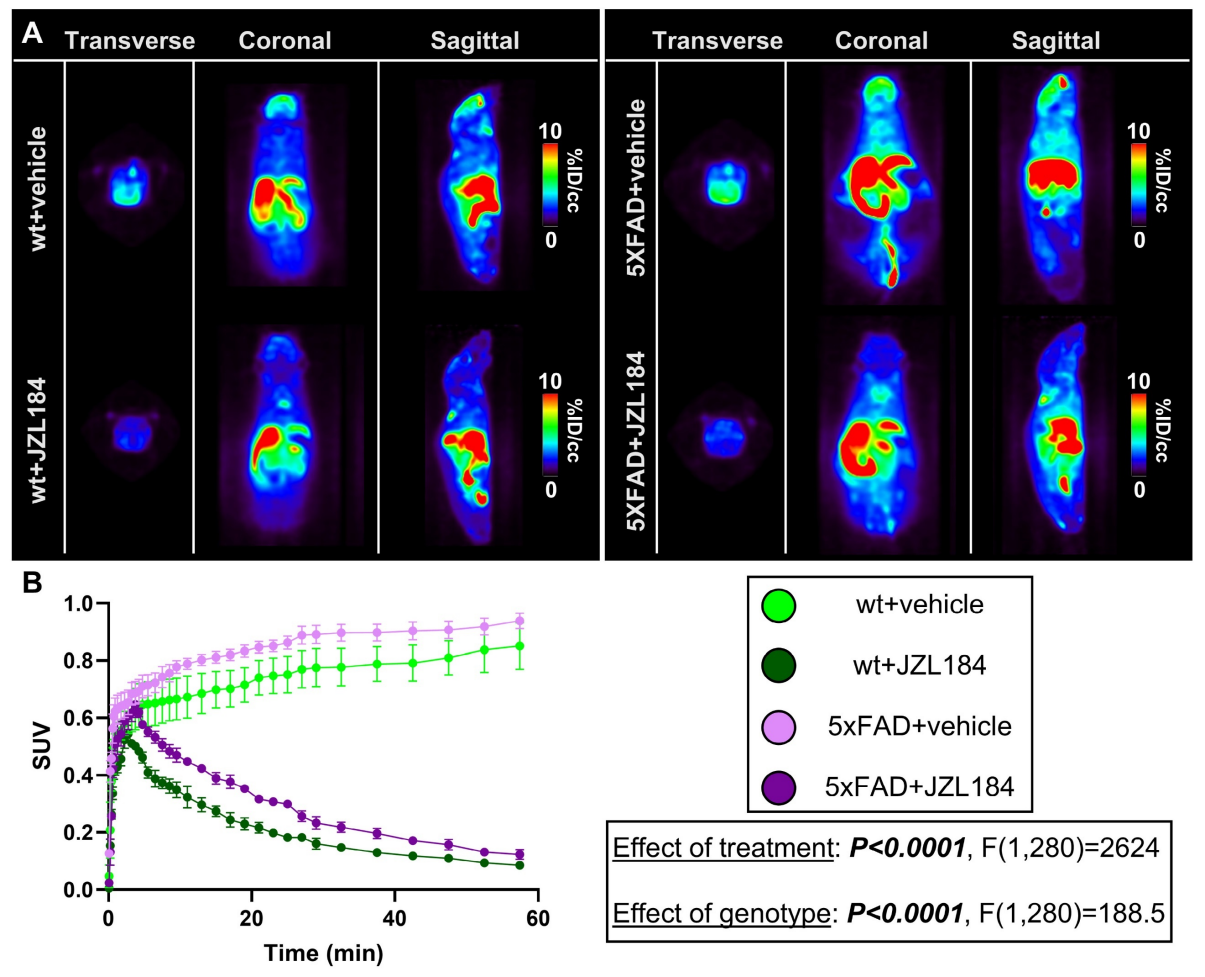
** A)** Representative summed [^18^F]MAGL-2102 PET images (0-60 min) and **B)** SUV curves demonstrating reduced MAGL availability following JZL184 treatment (MAGL inhibition) in 6-month wt and 5xFAD male mice (*n* = 3/genotype/treatment). 5xFAD mice exhibited higher MAGL availability than wt with or without JZL184. Mean+/-SEM. **Abbreviations:** ID/cc: injected dose per cubic centimetre; MAGL: monoacylglycerol lipase; PET: positron emission tomography; SUV: standardized uptake value; wt: wild-type.

To confirm target engagement of a MAGL therapeutic, we treated male adult wt and 5xFAD mice chronically with JZL184, a selective and irreversible MAGL inhibitor, and imaged with [^18^F]MAGL-2102 two days after ending treatment at 6-months of age (**Figure 6A**). Efficacy of JZL184 treatment to reduce AD hallmarks in 5xFAD mice has been previously described 11, and we employed a similar paradigm with JZL184 treatment to assess [^18^F]MAGL-2102 PET after MAGL inhibition. JZL184 treatment dramatically reduced MAGL availability (***P < 0.0001***, F(1,280) = 2624) with ~3.27x (wt) and ~2.52x (5xFAD) reductions in SUV at 20-minutes, and ~9.9x (wt) and ~7.65x (5xFAD) reductions at the final reading (**Figure 6B**). Significant genotype effects were also detected (***P < 0.0001***, F(1,280) = 188.5) with higher MAGL in 5xFAD vs wt mice, with or without treatment, supporting our observations in early-stage *App^NL-G-F^* mice (**Figure 2**). This data indicates the efficacy of [^18^F]MAGL-2102 imaging to monitor functional changes in MAGL levels and the clinical potential of this tracer for identifying individuals who could benefit the most from therapeutic MAGL inhibition.

## Discussion

The present study investigated the relationship of age, sex and AD pathology with the ECS, focused on PET imaging of the CB1 receptor and MAGL in a knock-in mouse model of AD (*App^NL-G-F^*). The endogenous APP expression patterns in *App^NL-G-F^* mice align Aβ deposition with normal aging events, facilitating characterization of interactive effects of age and sex with AD pathology. PET results were supplemented by autoradiography, immunostaining, western blots and a therapy study with the MAGL inhibitor JZL184, to confirm biological mechanisms of disease and the functional implications of the ECS as a biomarker of AD. Our PET results indicate early-stage sensitivity of CB1 signaling and endocannabinoid degradation in AD. [^18^F]FMPEP-*d*_2_ deficits start around plaque onset in *App^NL-G-F^* females, with compensatory increases in mid-stage pathology, and a significant loss in late-stage male and female* App^NL-G-F^* mice. Conversely, [^18^F]MAGL-2102 uptake increases early in AD in males, and returns to baseline with disease progression. Elucidating these distinctions is critical for disease mechanisms, therapeutic development, and for guiding future clinical research with [^18^F]FMPEP-*d*_2_ and [^18^F]MAGL-2102 in AD patients. To our knowledge this work represents the first study of CB1 and MAGL in *App^NL-G-F^* mice. Aβ plaque regional distribution and accumulation over age has been demonstrated in this mouse model in a previous PET imaging study with [^18^F]florbetaben, and is in line with our results 22.

### CB1 deficits in the early- and late-stages of *App^NL-G-F^* pathology

We observed decreased CB1 availability on [^18^F]FMPEP-*d*_2_ imaging in female *App^NL-G-F^* mice nearly brain-wide at 4-months (early-stage pathology) compared to male *App^NL-G-F^* and female wt, which may be related to higher susceptibility of females to AD 15; though CB1 protein was decreased at 4-months in *App^NL-G-F^* males *and* females. At 8-months of age we observed a plateau in which there were no significant differences in CB1 availability by sex or genotype, and a compensatory increase in hippocampal CB1 distribution and protein levels. CB1 is localized presynaptically and regulates neurotransmitter release, indicating potential contributions of hippocampal CB1 deficits and compensation to excitatory-inhibitory neuronal imbalance in AD 23-26, and the therapeutic potential of the ECS. At 12-months, CB1 availability was decreased again in *App^NL-G-F^* mice (m and f) notably in the frontal cortex, matching well with the protein levels, which were significantly reduced in the frontal cortex and striatum.

A previous PET imaging study aiming to investigate CB1 receptors in APP/PS1-21 AD mice showed significantly lower binding ratios of [^18^F]FMPEP-*d*_2_ in certain brain regions of 9- and 15-mo APP/PS1-21 mice, matching the results of our study; however, no differences in CB1 protein levels between any of the groups were detected 9. Another study investigated CB1 availability in AD in humans using the CB1 tracer [^18^F]MK-9470 10. The authors did not find any changes of CB1R availability in AD patients compared to healthy controls, nor a relationship with Aβ plaque density as measured by [^11^C]PIB PET. The inconsistencies with the results of our present study might be related to the use of a different tracer, or variation in the clinical presentation.

In other studies with [^18^F]MK-9470 it has been shown that CB1 availability increases over age specifically in healthy adult females 6,27. Increase in CB1 availability during aging has also been observed in the longitudinal study with [^18^F]FMPEP-*d*_2_ in 6- to 15-mo APP/PS1-21 and wt mice in parietotemporal cortex, hippocampus and cerebellum 9. We focused our statistical analyses on the effects of the AD genotype and sex; however, we did observe a notable decrease in CB1 availability in 8-mo female wt mice, compared to the 4- and 12-month timepoints. One possible explanation for this, and the discrepancy with the increased CB1 availability over age as reported in literature, is the influence of the estrous cycle and sex hormones on the ECS 14. Specifically, 8-month females are in the later stages of their reproductive age 28, and therefore sex hormones might have had a greater impact than at the earlier and later ages investigated, thereby reducing CB1 availability. Other reasons for the discrepancy could be different ages chosen (4 to 12-months (this study) vs. 6 to 15-months (Takkinen *et al.* 9)) and species differences between humans and rodents. Furthermore, CB1 mRNA and activity levels have been reported to decrease through adulthood 29,30 and in AD 8, indicating the dynamic effects age and sex can have on CB1 and the ECS.

The sexual dimorphism of the ECS and the influence of sex hormones, in particular estradiol, has long been established 14,31,32, and sex differences in CB1 receptor availability and expression have been reported in literature. A PET study with [^18^F]FMPEP-*d*_2_ in healthy humans for example showed a 41% higher signal in males compared to females brain-wide, with the largest effect in the posterior limbic cortex 33. Despite the lower female [^18^F]FMPEP-*d*_2_ availability at 8-months, we did not detect significant sex effects with PET in our control mice. This might be due to the small group size, or lack of controlling for the estrous cycle.

### Sex-dependent changes in MAGL availability and expression over pathological progression in *App^NL-G-F^* mice

With [^18^F]MAGL-2102, we demonstrate brain-wide increases of MAGL availability in male *App^NL-G-F^* mice at early-stage Aβ pathology, supported by increases in hippocampal MAGL protein. MAGL availability returns to baseline at mid- and late-stage pathology. This is congruent with recent findings in a study using the novel MAGL tracer (R)-[^11^C]YH132, where no significant differences in MAGL availability were found in the cortical region of 16-month mutant Tau-P301 L mice compared to wt 34. Late disease stage MAGL protein levels were increased specifically in female *App^NL-G-F^* cortex, and decreased in striatum of both sexes, with no hippocampal changes. Hippocampal MAGL mRNA, but not protein, decreases with age potentially in response to higher age-related MAGL activity 35.

In contrast to CB1, we did observe sex differences in MAGL availability. The sex differences were most pronounced at 8-months in both genotypes, but mostly significant for *App^NL-G-F^* mice only. This could be related to the different group sizes (*App^NL-G-F^* groups were larger (n = 7 per sex) than wt (n = 4)), or could be due to the AD pathology reinforcing the sex differences. A sex effect was also observed at 4-months, due to the higher MAGL availability in specifically male *App^NL-G-F^* mice. At 12-months no sex effect was observed, all groups had similar MAGL availability. This age-related sex effect in MAGL availability could, similarly as the decrease in CB1 availability in 8-months female wt mice, be related to the estrous cycle and reproductive age of the mice.

### PET imaging of MAGL inhibition *in vivo*

In an earlier study, MAGL inhibition with JZL184 was found to be neuroprotective and reduce Aβ in 5xFAD mice treated at early plaque stages 11. In our study, we used [^18^F]MAGL-2102 to confirm target engagement of JZL184 in wt and 5xFAD mice. MAGL availability was profoundly reduced in both genotypes after chronic MAGL inhibition compared to vehicle-treated controls. We also observed significant genotype effects with higher MAGL in male 5xFAD vs wt mice in treatment and control groups, which supports our previous data regarding elevated MAGL availability in early disease stages in males. Our data highlights a potential window for therapeutic MAGL inhibition in early-stage AD and that [^18^F]MAGL-2102 images functional change in MAGL levels, though this will be strengthened by validation in AD patient populations. Dynamic age, disease and sex alterations in MAGL will be critical to efficacy of MAGL inhibitor therapeutics 11,36, and clinical imaging with [^18^F]MAGL-2102 may prove fruitful for trial enrollment. The first MAGL PET imaging studies in humans were recently reported by Takahata *et al.* 37 using [^18^F]T-401 and further supports the urgency and interest of this class of tracers.

The effect of MAGL inhibition on CB1 has previously been investigated by Schlosburg *et al.* 38 in behavioural studies and through CB1 ligand binding in homogenates and autoradiography. It was found that CB1 receptors might be downregulated and/or desensitized in wildtype mice receiving JZL184 and in MAGL knockout mice. For future studies, it might be interesting to investigate CB1 availability via [^18^F]FMPEP-*d*_2_ imaging in mice receiving a MAGL inhibitor, especially in a mouse model of AD.

### Study limitations

For both CB1 and MAGL, some discrepancies between target availability (assessed by PET imaging) and protein expression (assessed by western blot) were observed in our studies. While it certainly validates the results when trends in availability and expression align, they reflect different aspects in the biological process and differences can be expected. Especially for CB1, the radiotracer will most likely only bind to the active receptor on the cell membrane and might not be able to penetrate the cell and bind to internally located CB1 reservoirs 39. Western blotting, however, assesses relative protein amount regardless of its cellular location and functional status. Another CB1 inverse agonist radiopharmaceutical and structural analog to [^18^F]FMPEP-*d*_2_, [^11^C]MePPEP, has been demonstrated to be displaced by CB1 inverse agonists but only at low potency by agonists, which the authors concluded may in part be due to a significant CB1 receptor reserve 40,41. Our observed CB1 compensation in AD mice, and AD-related dynamics in availability per protein, support the notion of a CB1 protein reserve not captured by PET imaging which is involved in AD progression.

It has also been suggested that a higher protein expression could be a counterregulatory response to functional desensitization of CB1, which influences the radioligand binding 42. Another aspect could be differences in time of assessment. CB1 protein changes, for example, may better align with PET imaging than MAGL because the mice were sacrificed immediately after the FMPEP scan, whereas MAGL imaging was a week prior. Further technical aspects leading to discrepancies could be overestimation of ligand binding in the PET images due to potential spill-over 39 and potential differences in the mouse brain atlas alignment conducted for PET analysis vs. brain sub-dissection for western blotting.

Further limitations of the current study include a low sample size for age*sex*pathology interactive statistics, and future work could further these statistical analyses, expand ECS imaging targets to FAAH and CB2, and include comparisons to longitudinal Aβ and synaptic PET. Also pertinent for sex differences would be controlling for estrous cycle, considering interactions of ECS with sex hormones 14. Another limitation of this study is the analysis of the PET data, which was simplified by comparing the AUCs of the TACs. Full quantitative analysis including determination of volume of distribution (V_T_) and binding potential (BP_ND_) would increase the accuracy but faces some practical challenges in PET imaging of mice due to the limited blood volume available for arterial sampling 43. We are currently exploring the use of a population-based input function for the imaging of [^18^F]MAGL-2102/[^18^F]FMPEP-*d*_2_ in rodents which could be a viable alternative and allow more accurate analysis of the PET data in the future. It should be also noted that the present study was not longitudinal and only limited comparisons can be drawn among groups of different ages. Both radiopharmaceuticals are currently translated for human PET imaging studies at our laboratory.

## Conclusions

Herein, we describe stage-related ECS deficits in an AD mouse model: CB1 levels with [^18^F]FMPEP-*d*_2_ imaging are lost at the early-stage prominently in females, undergo a compensatory increase, and are deficient again at the late-stage pathology in both sexes; [^18^F]MAGL-2102 availability increases only in the early-stage pathology in males. [^18^F]MAGL-2102 imaging also demonstrates functional change following therapeutic MAGL inhibition. Understanding the sex, age and disease-related trends in the ECS is important for the biological basis of Alzheimer's disease as well as in the design of cannabinoid therapeutic paradigms. Future work to establish the relationship between CB1 and MAGL bioavailability with Aβ and/or tau PET could provide insights for testing and personalizing the therapeutic engagement of endocannabinoid treatment in AD, and will be used to guide our upcoming human PET imaging studies with these radiopharmaceuticals.

## Supplementary Material

Supplementary methods, figures and tables.

## Figures and Tables

**Table 1:** Specifications of the mice that underwent [^18^F]FMPEP-*d*_2_ scans.

Entry	Age	Genotype	Sex	Weight (g)	ID (MBq)	Mass (nmol/kg)	n
1	4 mo	*App^NL-G-F^*	f	18.8-20.9	2.03-5.16	0.37-2.65	4
2	4 mo	*App^NL-G-F^*	m	26.7-29.5	3.26-5.88	0.37-1.93	4
3	4 mo	C57BL/6	f	18.3-21.0	2.68-6.55	0.38-7.95	4
4	4 mo	C57BL/6	m	24.0-28.7	3.92-5.96	0.44-5.89	4
5	8 mo	*App^NL-G-F^*	f	20.8-23.1	2.07-5.60	0.58-1.76	6
6	8 mo	*App^NL-G-F^*	m	28.0-31.8	2.03-5.70	0.38-0.72	5
7	8 mo	C57BL/6	f	23.9-26.1	3.27-5.31	0.49-1.77	3
8	8 mo	C57BL/6	m	28.5-34.3	4.09-4.26	0.27-1.64	3
9	12 mo	*App^NL-G-F^*	f	23.9-25.2	3.93-4.89	0.50-1.66	4
10	12 mo	*App^NL-G-F^*	m	28.7-32.0	4.35-5.94	0.65-0.88	4
11	12 mo	C57BL/6	f	23.4-29.2	2.36-5.44	0.22-1.60	5
12	12 mo	C57BL/6	m	31.5-36.9	4.72-6.37	0.49-1.39	4

**Abbreviations:** f: female; ID: injected dose; m: male; mo: months

**Table 2:** Specifications of the mice that underwent [^18^F]MAGL-2102 scans.

Entry	Age	Genotype	Sex	Weight (g)	ID (MBq)	Mass (nmol/kg)	n
1	4 mo	*App^NL-G-F^*	f	18.4-22.0	2.48-5.00	0.36-15.13	4
2	4 mo	*App^NL-G-F^*	m	26.5-30.8	2.62-6.89	0.25-10.23	4
3	4 mo	C57BL/6	f	18.2-21.9	2.07-4.84	0.38-11.66	4
4	4 mo	C57BL/6	m	23.4-28.8	2.94-6.21	0.26-13.28	4
5	8 mo	*App^NL-G-F^*	f	20.5-23.3	2.73-5.95	0.85-4.88	7
6	8 mo	*App^NL-G-F^*	m	28.5-32.4	3.92-6.94	0.51-4.51	7
7	8 mo	C57BL/6	f	24.7-27.7	4.20-6.63	0.58-4.44	4
8	8 mo	C57BL/6	m	28.7-35.5	4.30-5.88	0.44-3.40	4
9	12 mo	*App^NL-G-F^*	f	23.8-26.9	4.75-6.74	1.03-4.41	4
10	12 mo	*App^NL-G-F^*	m	29.5-31.9	3.87-6.25	0.72-7.81	4
11	12 mo	C57BL/6	f	26.3-29.3	3.79-6.30	0.81-3.72	4
12	12 mo	C57BL/6	m	31.8-37.7	3.24-8.27	0.59-3.96	4

**Abbreviations:** f: female; ID: injected dose; m: male; mo: months

**Table 3:** AUC data and statistics of the 4-month [^18^F]FMPEP-*d*_2_ scans demonstrating widespread reduced CB1 availability in female *App^NL-G-F^* mice.

Region	Two-way ANOVA (dF: 1,12)	Holm-Šídák post-hoc, (AUC mean±SEM)
Whole brain	Genotype: F = 2.252, P = 0.1593 Sex: F = 3.677 *P = 0.0793* Genotype*sex: F = 6.226, ***P = 0.0282***	wt-f (161.9±5.4) vs. NLGF-f (138.9±5.2): ***P = 0.0304*** wt-m (158.5±6.9) vs. NLGF-m (164.3±5.4): P = 0.4953 wt-f vs. wt-m: P = 0.6901; NLGF-f vs. NLGF-m: ***P = 0.0176***
Caudate putamen	Genotype: F = 1.723, P = 0.2138 Sex: F = 4.822; ***P = 0.0485*** Genotype*sex: F = 7.378, ***P = 0.0187***	wt-f (195.3±6.7) vs. NLGF-f (166.5±6.4): ***P = 0.0291*** wt-m (191.6±8.1) vs. NLGF-m (201.7±7.3): P = 0.3406 wt-f vs. wt-m: P = 0.7194; NLGF-f vs. NLGF-m: ***P = 0.0092***
Frontal cortex	Genotype: F = 4.392, *P = 0.0580* Sex: F = 4.567, *P = 0.0539* Genotype*sex: F = 7.224, ***P = 0.0197***	wt-f (163.0±4.9) vs. NLGF-f (136.9±3.5): ***P = 0.0109*** wt-m (160.0±8.1) vs. NLGF-m (163.2±4.1): P = 0.6828 wt-f vs. wt-m: P = 0.7037; NLGF-f vs. NLGF-m: ***P = 0.0103***
Hippocampus	Genotype: F = 0.9994, P = 0.3372 Sex: F = 4.407, *P = 0.0576* Genotype*sex: F = 6.354, ***P = 0.0269***	wt-f (179.6±6.9) vs. NLGF-f (154.9±7.1): ***P = 0.0561*** wt-m (176.6±7.1) vs. NLGF-m (187.3±7.0): P = 0.3033 wt-f vs. wt-m: P = 0.7709; NLGF-f vs. NLGF-m: ***P = 0.0134***
Parietal-temporal cortex	Genotype: F = 3.954, *P = 0.0701* Sex: F = 3.668, *P = 0.0796* Genotype*sex: F = 4.251, *P = 0.0616*	wt-f (146.2±4.2); wt-m (145.5± 6.9) NLGF-f (125.5± 4.6); NLGF-m (145.8± 4.4)
Occipital cortex	Genotype: F = 1.044, P = 0.3271 Sex: F = 7.004, ***P = 0.0213*** Genotype*sex: F = 2.466, P = 0.1423	wt-f (144.3±4.6) vs. wt-m (150.5±7.8): P = 0.4614 NLGF-f (129.4±4.3) vs. NLGF-m (153.6±5.5): ***P = 0.0228***
Medulla	Genotype: F = 0.6595, P = 0.4326 Sex: F = 3.664, *P = 0.0797* Genotype*sex: F = 4.492, *P = 0.0556*	wt-f (139.4±4.5); wt-m (138.3±6.7) NLGF-f: (123.0±5.7); NLGF-m (145.6±5.4)
Midbrain	Genotype: F = 3.062, P = 0.1057 Sex: F = 1.333, P = 0.2708 Genotype*sex: F = 6.823, ***P = 0.0227***	Wt-f (194.2±7.3) vs. NLGF-f (164.0±8.3): ***P = 0.0188*** Wt-m (184.1±6.0) vs. NLGF-m (190.1±5.9): P = 0.5534 wt-f vs. wt-m: P = 0.3230; NLGF-f vs. NLGF-m: ***P = 0.0409***
Pons	Genotype: F = 0.9826, P = 0.3411 Sex: F = 2.069, P = 0.1759 Genotype*sex: F = 6.343, ***P = 0.0270***	wt-f (161.9±6.5) vs. NLGF-f (140.7±5.8): *P = 0.0569* wt-m (155.4±5.5) vs. NLGF-m (164.6±6.4): P = 0.3014 wt-f vs. wt-m: P = 0.4597; NLGF-f vs. NLGF-m: ***P = 0.0320***
Thalamus	Genotype: F = 3.447, *P = 0.0881* Sex: F = 0.9448 P = 0.3502 Genotype*sex: F = 8.313, ***P = 0.0137***	wt-f (198.6±7.0) vs. NLGF-f (166.1±8.0): ***P = 0.0115*** wt-m (185.5±5.5) vs. NLGF-m (192.6±6.8): P = 0.4818 wt-f vs. wt-m: P = 0.4597; NLGF-f vs. NLGF-m: ***P = 0.0365***
Cerebellum	Genotype: F = 0.1208, P = 0.7342 Sex: F = 8.471 ***P = 0.0131*** Genotype*sex: F = 3.551, *P = 0.0840*	wt-f (137.4±3.6) vs. wt-m (142.9±6.9): P = 0.4820 NLGF-f (125.6±4.1) vs. NLGF-m (151.0±5.8): ***P = 0.0107***

**Abbreviations:** AUC: area under the curve; CB1: cannabinoid receptor 1; dF: degrees of freedom; f: female; m: male; NLGF: *App^NL-G-F^*; wt: wild-type.

**Table 4:** AUC data and statistics of the 12-month [^18^F]FMPEP-*d*_2_ scans demonstrating reduced CB1 availability in female and male *App^NL-G-F^* mice, notably in the frontal cortex.

Region	Two-way ANOVA (dF: 1,13)	Holm-Šídák post-hoc, (AUC mean±SEM)
Whole brain	Genotype: F = 4.563, *P = 0.0523* Sex: F = 0.0095, P = 0.9238 Genotype*sex: F = 0.4367, P = 0.5203	wt-f (166.3±8.8); wt-m (160.9±5.0) NLGF-f (146.4±2.1); NLGF-m (150.4±8.4)
Caudate putamen	Genotype: F = 2.204, P = 0.1615 Sex: F = 0.0459, P = 0.8336 Genotype*sex: F = 0.6584, P = 0.4317	wt-f (199.1±11.3); wt-m (193.1±6.6) NLGF-f (176.0±3.2); NLGF-m (179.3± 11.0)
Frontal cortex	Genotype: F = 6.022, ***P = 0.0290*** Sex: F = 0.2044, P = 0.6587 Genotype*sex: F = 0.2168, P = 0.6492	wt-f (173.6± 8.8) vs. NLGF-f (152.1± 3.0): P = 0.1045 wt-m (166.9± 6.0) vs. NLGF-m (152.2± 8.5): P = 0.1045
Hippocampus	Genotype: F = 2.870, P = 0.1141 Sex: F = 0.0252, P = 0.8763 Genotype*sex: F = 0.8594, P = 0.3708	wt-f (183.7±9.4); wt-m (177.6±4.2) NLGF-f (162.9±3.6); NLGF-m (171.5±10.5)
Parietal-temporal cortex	Genotype: F = 3.117, P = 0.1009 Sex: F = 0.0973, P = 0.7600 Genotype*sex: F = 0.3957, P = 0.5402	wt-f (151.8± 7.6); wt-m (149.7± 6.3) NLGF-f (135.3± 3.0); NLGF-m (141.8± 8.5)
Occipital cortex	Genotype: F = 1.741, P = 0.2098 Sex: F = 0.5179, P = 0.4845 Genotype*sex: F = 0.1402, P = 0.7141	wt-f (153.5± 7.6); wt-m (156.4± 7.8) NLGF-f (139.3± 4.0); NLGF-m (148.5± 12.1)
Medulla	Genotype: F = 5.456, ***P = 0.0362*** Sex: F = 0.0351, P = 0.8542 Genotype*sex: F = 0.1353, P = 0.7189	wt-f (139.6± 7.9) vs. NLGF-f (123.0± 1.5): P = 0.1374 wt-m (138.5± 4.2) vs. NLGF-m (126.4± 6.8): P = 0.1981
Midbrain	Genotype: F = 3.962, *P = 0.0680* Sex: F = 0.0893, P = 0.7699 Genotype*sex: F = 0.4256, P = 0.5255	wt-f (189.5± 10.4); wt-m (181.7± 5.3) NLGF-f (167.8± 2.0); NLGF-m (170.7± 9.6)
Pons	Genotype: F = 5.740, ***P = 0.0323*** Sex: F = 0.0748, P = 0.7888 Genotype*sex: F = 0.5333, P = 0.4782	wt-f (161.7± 9.4) vs. NLGF-f (140.0± 2.5): *P = 0.0799* wt-m (154.7± 3.1) vs. NLGF-m (143.2± 7.5): P = 0.2717
Thalamus	Genotype: F = 4.273, *P = 0.0592* Sex: F = 0.0537, P = 0.8204 Genotype*sex: F = 0.5977, P = 0.4533	wt-f (192.9±11.0); wt-m (184.5±5.3) NLGF-f (169.1±2.0); NLGF-m (173.6±9.5)
Cerebellum	Genotype: F = 3.256, *P = 0.0944* Sex: F = 0.4137, P = 0.5313 Genotype*sex: F = 0.0329, P = 0.8588	wt-f (142.5±7.2); wt-m (145.6±4.4) NLGF-f (129.3±1.8); NLGF-m (134.8±9.6)

**Abbreviations:** AUC: area under the curve; CB1: cannabinoid receptor 1; dF: degrees of freedom; f: female; m: male; NLGF: *App^NL-G-F^*; wt: wild-type.

**Table 5:** AUC data and statistics of the 4-month [^18^F]MAGL-2102 scans demonstrating higher MAGL availability in male *App^NL-G-F^* mice.

Region	Two-way ANOVA (dF: 1,12)	Holm-Šídák post-hoc, (AUC mean±SEM)
Whole brain	Genotype: F = 5.270, ***P = 0.0405*** Sex: F = 5.749, ***P = 0.0337*** Genotype*sex: F = 0.1576, P = 0.6984	wt-f (100.2±9.8) vs. NLGF-f (111.9±2.9): P = 0.3668 wt-m (112.5±3.7) vs. NLGF-m (129.0±5.6): P = 0.1558 wt-f vs. wt-m: P = 0.3318; NLGF-f vs. NLGF-m: P = 0.1381
Caudate putamen	Genotype: F = 5.860, ***P = 0.0323*** Sex: F = 7.311, ***P = 0.0192*** Genotype*sex: F = 1.208, P = 0.2932	wt-f (133.6±3.5) vs. NLGF-f (139.6±4.2): P = 0.6013 wt-m (140.9±4.3) vs. NLGF-m (156.7±5.7): *P = 0.0562* wt-f vs. wt-m: P = 0.4797; NLGF-f vs. NLGF-m: ***P = 0.0390***
Frontal cortex	Genotype: F = 3.772, *P = 0.0760* Sex: F = 5.264, ***P = 0.0406*** Genotype*sex: F = 1.659, P = 0.2221	wt-f (134.4±3.2) vs. wt-m (139.9±6.6): P = 0.7402 NLGF-f (137.9±4.7) vs. NLGF-m (157.5±6.6): *P = 0.0519*
Hippocampus	Genotype: F = 5.272, ***P = 0.0405*** Sex: F = 6.128, ***P = 0.0292*** Genotype*sex: F = 0.8416, P = 0.3770	wt-f (114.2±5.7) vs. NLGF-f (120.9±4.1): P = 0.5760 wt-m (121.8±4.2) vs. NLGF-m (137.5±5.4): *P = 0.0828* wt-f vs. wt-m: P = 0.4990; NLGF-f vs. NLGF-m: *P = 0.0660*
Parietal-temporal cortex	Genotype: F = 4.323, *P = 0.0597* Sex: F = 4.051, *P = 0.0671* Genotype*sex: F = 0.1639, P = 0.6927	wt-f (103.6±7.4); wt-m (113.3±6.4) NLGF-f (113.7±3.2); NLGF-m (128.2±6.1)
Occipital cortex	Genotype: F = 4.642, *P = 0.0522* Sex: F = 3.979, *P = 0.0693* Genotype*sex: F = 0.0179, P = 0.8959	wt-f (87.7±6.8); wt-m (96.9±5.0) NLGF-f (97.7±1.3); NLGF-m (108.2±5.0)
Medulla	Genotype: F = 0.1486, P = 0.7066 Sex: F = 0.0696, P = 0.7964 Genotype*sex: F = 1.383, P = 0.2624	wt-f (77.3±5.2); wt-m (74.5±2.8) NLGF-f (74.9±0.9); NLGF-m (79.3±1.5)
Midbrain	Genotype: F = 4.374, *P = 0.0584* Sex: F = 4.140, *P = 0.0646* Genotype*sex: F = 0.0428, P = 0.8396	wt-f (93.5±7.3); wt-m (100.8±2.2) NLGF-f (101.0±2.2); NLGF-m (110.0±1.3)
Pons	Genotype: F = 0.0103, P = 0.9210 Sex: F = 0.0103, P = 0.9210 Genotype*sex: F = 2.506, P = 0.1394	wt-f (86.1±6.8); wt-m (79.9±2.6) NLGF-f (80.7±1.3); NLGF-m (86.1±0.3)
Thalamus	Genotype: F = 5.907, ***P = 0.0317*** Sex: F = 7.802, ***P = 0.0162*** Genotype*sex: F = 1.055, P = 0.3247	wt-f (113.6±3.5) vs. NLGF-f (118.8±3.9): P = 0.5652 wt-m (120.1±2.7) vs. NLGF-m (132.7±4.3): *P = 0.0608* wt-f vs. wt-m: P = 0.4155; NLGF-f vs. NLGF-m: ***P = 0.0382***
Cerebellum	Genotype: F = 3.193, *P = 0.0992* Sex: F = 5.293, ***P = 0.0402*** Genotype*sex: F = 0.2866, P = 0.6022	wt-f (78.4±3.1) vs. wt-m (83.3±2.8): P = 0.4159 NLGF-f (81.9±2.1) vs. NLGF-m (89.8±3.0): P = 0.1314

**Abbreviations:** AUC: area under the curve; dF: degrees of freedom; f: female; m: male; MAGL: monoacylglycerol lipase; NLGF: *App^NL-G-F^*; wt: wild-type.

**Table 6:** AUC data and statistics of the 8-month [^18^F]MAGL-2102 scans demonstrating widespread lower MAGL availability in female, compared to male, *App^NL-G-F^* mice.

Region	Two-way ANOVA (dF: 1,18)	Holm-Šídák post-hoc, (AUC mean±SEM)
Whole brain	Genotype: F = 2.659, P = 0.1203 Sex: F = 12.22, ***P = 0.0026*** Genotype*sex: F = 0.5253, P = 0.4779	wt-f (106.9±5.3) vs. wt-m (119.9±2.6): P = 0.1892 NLGF-f (95.9±4.3) vs. NLGF-m (115.6±4.4): ***P = 0.0051***
Caudate putamen	Genotype: F = 2.453, P = 0.1347 Sex: F = 10.92, ***P = 0.0039*** Genotype*sex: F = 0.2938, P = 0.5944	wt-f (134.5±6.3) vs. wt-m (151.6±3.6): P = 0.1908 NLGF-f (121.4±5.5) vs. NLGF-m (145.2±6.2): ***P = 0.0101***
Frontal cortex	Genotype: F = 4.577, ***P = 0.0464*** Sex: F = 9.504, ***P = 0.0064*** Genotype*sex: F = 0.4538, P = 0.5091	wt-f (136.7±7.8) vs. NLGF-f (119.3±5.6): P = 0.1204 wt-m (151.6±3.6) vs. NLGF-m (142.5±5.6): P = 0.5291 wt-f vs. wt-m: P = 0.2747; NLGF-f vs. NLGF-m: ***P = 0.0119***
Hippocampus	Genotype: F = 2.361, P = 0.1418 Sex: F = 10.39, ***P = 0.0047*** Genotype*sex: F = 0.1042, P = 0.7505	wt-f (113.7±6.8) vs. wt-m (130.8±4.3): P = 0.1640 NLGF-f (102.8±5.3) vs. NLGF-m (123.7±5.4): ***P = 0.0174***
Parietal-temporal cortex	Genotype: F = 3.662, *P = 0.0717* Sex: F = 9.833, ***P = 0.0057*** Genotype*sex: F = 0.5028, P = 0.4874	wt-f (109.9±7.0) vs. wt-m (122.8±3.8): P = 0.2701 NLGF-f (96.0±4.3) vs. NLGF-m (116.4±5.0): ***P = 0.0102***
Occipital cortex	Genotype: F = 1.926, P = 0.1821 Sex: F = 12.27, ***P = 0.0025*** Genotype*sex: F = 1.510, P = 0.2349	wt-f (92.3±5.9) vs. wt-m (102.1±2.5): P = 0.3133 NLGF-f (81.0±3.2) vs. NLGF-m (101.5±4.3): ***P = 0.0020***
Medulla	Genotype: F = 0.0185, P = 0.8933 Sex: F = 14.92, ***P = 0.0011*** Genotype*sex: F = 2.481, P = 0.1327	wt-f (67.4±1.5) vs. wt-m (73.8±2.9): P = 0.3089 NLGF-f (63.3±2.7) vs. NLGF-m (78.6±2.6): ***P = 0.0005***
Midbrain	Genotype: F = 1.033, P = 0.3230 Sex: F = 14.97, ***P = 0.0011*** Genotype*sex: F = 0.1432, P = 0.7096	wt-f (90.4±3.6) vs. wt-m (104.5±2.4): *P = 0.0824* NLGF-f (84.8±4.0) vs. NLGF-m (101.9±3.7): ***P = 0.0049***
Pons	Genotype: F = 0.2078, P = 0.6539 Sex: F = 18.20, ***P = 0.0005*** Genotype*sex: F = 1.234, P = 0.2813	wt-f (71.1±1.9) vs. wt-m (79.9±2.3): P = 0.1230 NLGF-f (66.8±2.8) vs. NLGF-m (81.7±2.5): ***P = 0.0006***
Thalamus	Genotype: F = 1.947, P = 0.1799 Sex: F = 12.92, ***P = 0.0021*** Genotype*sex: F = 0.0277, P = 0.8697	wt-f (109.0±6.3) vs. wt-m (127.6±2.9): *P = 0.0890* NLGF-f (100.5±5.0) vs. NLGF-m (120.9±5.1): ***P = 0.0118***
Cerebellum	Genotype: F = 0.2157, P = 0.6479 Sex: F = 19.20, ***P = 0.0004*** Genotype*sex: F = 3.780, *P = 0.0677*	wt-f (76.9±2.2) vs. wt-m (84.5±2.4): P = 0.2672 NLGF-f (69.4±2.8) vs. NLGF-m (89.2±3.2): ***P = 0.0001***

**Abbreviations:** AUC: area under the curve; dF: degrees of freedom; f: female; m: male; MAGL: monoacylglycerol lipase; NLGF: *App^NL-G-F^*; wt: wild-type.

## Abbreviations

2-AG: 2-arachidonoylglycerol
AD: Alzheimer's disease
AEA: anandamide
ARG: autoradiography
AUC: area under the curve
CB1: cannabinoid receptor 1
CB2: cannabinoid receptor 2
ECS: endocannabinoid system
FAAH: fatty acid amide hydrolase
GCL: granular cell layer
IF: immunofluorescence
MAGL: monoacylglycerol lipase
PET: positron emission tomography
PCL: pyramidal cell layer
SUV: standardized uptake value
TAC: time activity curve
wt: wild-type
