# Supplementary material for: Sex- and age-specific sensitivities of the endocannabinoid system in Alzheimer's disease revealed by PET imaging with [18F]FMPEP-d2 and [18F]MAGL-2102
Source: Theranostics. 2025 Feb 18;15(8):3368–85. doi: 10.7150/thno.106592 (PMC11905134; doi:10.7150/thno.106592)
Supplement: Supplementary file 1 — Supplementary methods, figures and tables. [file thnov15p3368s1.pdf]

Supplementary materials

Contents

Supplementary methods 2

General 2

Animals 3

Radiochemistry 4

Supplementary tables 6

Supplementary figures 9

Supplementary methods

General

The reference standard (*3R,5R*)-5-[3-(dideuterofluoromethoxy)phenyl]-3-[(*R*)-1-phenylethylamino]-1-(4-trifluoromethylphenyl)pyrrolidine-2-one (FMPEP-d_2) was custom-synthesized by PharmaSynth AS (Tartu, Estonia). The precursors PPEP and ditosylmethane-d_2 were custom-synthesized by Toronto Research Chemicals (Toronto, Canada). Primary antibodies were obtained from Abcam Inc. (Waltham, USA). All other chemicals and reagents were obtained from commercial vendors and were used as received without further purification. MAGL-2102 precursor and reference standard were custom-synthesized by Toronto Research Chemicals (Toronto, Canada).

[^{18}F]Fluoride was produced with a MC-17 cyclotron (Scanditronix, Sweden) from an oxygen-18 enriched water target. Analytical HPLC was performed on two systems. For [^{18}F]FMPEP-d_2, a 1260 Infinity II quaternary gradient HPLC system (Agilent; Santa Clara, California, US) was used which was connected in series with a radioactivity detector (Ludlum; Sweetwater, Texas, US). Analysis was performed using the Agilent Data Analysis chromatography software (Agilent; Santa Clara, California, US). [^{18}F]MAGL-2102 was analyzed on a system comprised of a high-pressure isocratic pump (LC-20AT, Shimadzu Inc.; Kyoto, Japan) equipped with a variable wavelength ultraviolet (UV) detector (λ = 254 nm, SPD-20A, Shimadzu Inc.) connected in series with a radioactivity detector (Frisk-tech, Bicron; Torrington, Connecticut, US). Analysis was performed using PowerChrom chromatography software (eDAQ Pty Ltd.; Colorado Springs, Colorado, US). Radiochemistry was performed in lead-shielded hot cells in a laboratory designed to handle radioactive material. The work was performed in

accordance with the radiation protection guidelines and regulations from the Canadian Nuclear Safety Commission and internal safety policies and procedures.

Animals and methodology for Thioflavin-S analysis

An additional eighteen $App^{NL\text{-}G\text{-}F}$ mice (n = 3/sex/age) were utilized for characterization of β-amyloid plaque staging (**Figure S1**). These mice were transcardially perfused with 1xPBS followed by 4% paraformaldehyde, and the brains were sectioned coronally at 40 µm on a microtome. Free floating sections from the paraformaldehyde perfused mice were sampled for frontal cortex, striatum and hippocampus and stained for Thioflavin-S and DAPI. Sections were mounted on slides and cover slipped. Thioflavin-S+ plaques were quantified manually in ImageJ and normalized to area. When applicable, all mice were randomized to cohorts and experimenters were blinded.

Radiochemistry

[^{18}F]FMPEP-d_2 synthesis

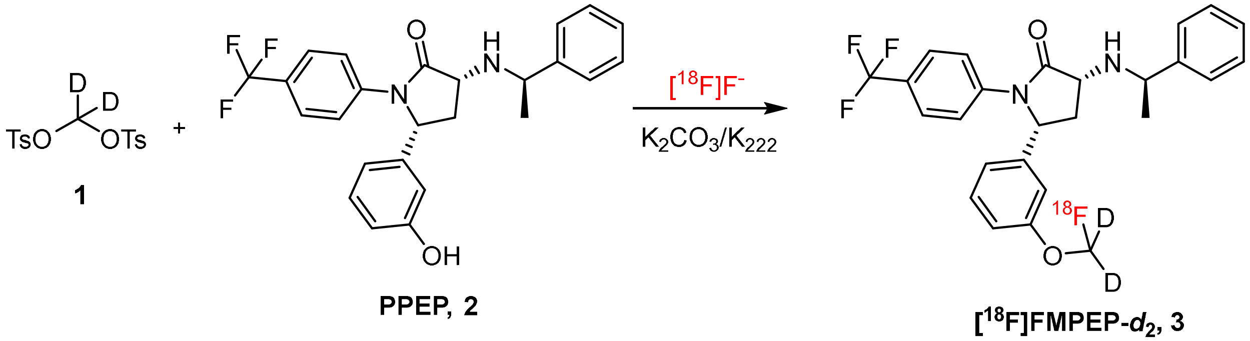

[^{18}F]FMPEP-d_2 was synthesized as previously described [17] and was obtained with a RCY of 8±1% (dc), a A_m of 322±101 GBq/µmol (EOS) and a RCP >95% in a synthesis time of ~70 min (n = 8) in a solution of 10% EtOH in saline (0.9%; pH 5.6).

[^{18}F]MAGL-2102 synthesis

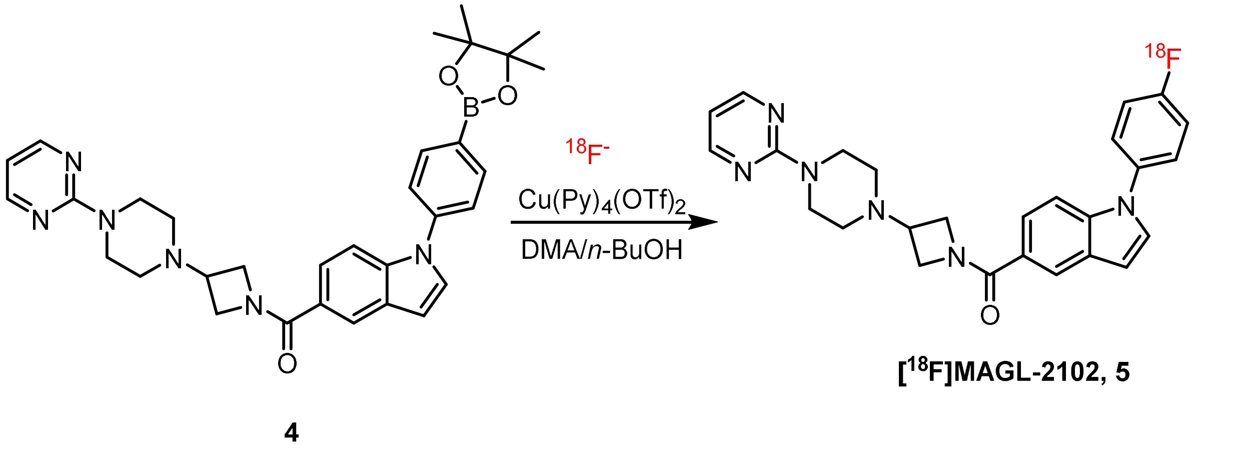

[^{18}F]MAGL-2102 was prepared using an automated GE TRACERlab FX2 N synthesis module according to a modified literature procedure [13]: No-carrier-added [^{18}F]fluoride (10-25 GBq) was produced from oxygen-18 enriched water via the $^{18}O(p,n)^{18}F$ nuclear reaction

(Scanditronix MC-17 cyclotron). The aqueous solution containing [^{18}F]fluoride was transferred to the GE TRACERlab FX2 N synthesis module and trapped on a ^{18}F separation cartridge (45 mg PS-HCO_3 cartridge; Synthra, Germany). The [^{18}F]fluoride was eluted with 1 mL of a solution of Et_4NHCO_3 (0.5 mg/mL) in MeOH into the reactor and was azeotropically dried at 85-110 °C under addition of 1 mL CH_3CN with a N_2 flow and vacuum. A mixture of (3-(4-(pyrimidin-2-yl)piperazin-1-yl)azetidin-1-yl)(1-(4-(4,4,5,5-tetramethyl-1,3,2-dioxaborolan-2-yl)phenyl)-1H-indol-5-yl)methanone **4** (3 mg) and $Cu(Py)_2OTf_2$ (7 mg) co-dissolved in 400 µL DMAc and 200 µL *n*-BuOH was added to the dry [^{18}F]fluoride, and the reaction was heated for 20 min at 110 °C. After dilution with 3 mL of 20% CH_3CN in water, the mixture was loaded onto the HPLC loop using N_2 pressure and guided with a fluid detector. The product was purified using a semi-preparative HPLC column (Waters XSelect HSS T3 OBDTM (250x10, 5µ) + Guard Column) and eluted with a solvent mixture of 40:60 CH3CN/0.1M ammonium formate at a flow rate of 6 mL/min. The eluent was monitored by UV (λ = 254 nm) and a radioactivity detector. The HPLC fraction containing the product (t_R ~ 25 min) was collected, diluted with 25 mL water and 2 mL of 1N $NaHCO_3$, and passed over a Sep-Pak tC18 plus short cartridge. The cartridge was washed with 10 mL sterile water and [^{18}F]MAGL-2102 was eluted with 1 mL ethanol followed by 9 mL 0.9% sodium chloride for injection, resulting in a solution of 10% EtOH (v/v) in saline. [^{18}F]MAGL-2102 was obtained with a RCY of 22±8% (dc), a A_m of 271±125 GBq/µmol (EOS) and a RCP >95% in a synthesis time of ~75 min (n = 12). An aliquot of the product was measured after each synthesis by analytical HPLC using a Prodigy ODS-prep C18 10µ 250x4.6 mm column, a flow rate of 2 mL/min and CH_3CN/0.1M ammonium formate in water 50:50. The identity of the product was confirmed by co-injection with the unlabelled reference standard.

Supplementary tables

Table S1: Immunofluorescence and Western Blot Antibodies and Detection Reagents

Antibody	**Vendor**	**Reference #**	**Dilution**
rabbit anti-CB1	Abcam	ab23703	1:200 (IF), 1:1000 (WB)
rabbit anti-MAGL	Abcam	ab24701	1:100 (both)
mouse anti-SNAP25	Abcam	ab66066	1:1000 (WB)
goat anti-rabbit Alexafluor568	Invitrogen	A11011	1:200 (IF)
HRP-goat anti-rabbit IgG	Jackson Immunoresearch Labs	111-035-008	1:10000 (WB)
HRP-goat anti-mouse IgG+IgM	Jackson Immunoresearch Labs	115-035-044	1:10000 (WB)
mouse anti-β-actin	Invitrogen	AM4302	1:5000 (WB)
DAPI	Roche Diagnostics	10236276001	1:5000 (IF)
Thioflavin-S	Sigma-Aldrich	T1892	1% wt/vol in ddH2O (IF)

*CB1: Cannabinoid Receptor 1; HRP: horseradish peroxidase; IF: immunofluorescence; MAGL: monoacylglycerol lipase; SNAP-25: Synaptosomal-Associated Protein, 25 kDa; WB: western blot

Table S2: AUC data and statistics (2-way ANOVA with Šídák's multiple comparisons test) of the 8mo [^{18}F]FMPEP-d_2 scans

**Region**	**Two-way ANOVA** (dF: 1,13)	**Holm-Šídák post-hoc, (AUC mean±SEM)**
Whole brain	Genotype: F = 0.04646; P = 0.8327<br>Sex: F = 0.8708; P = 0.3677<br>Genotype*sex: F = 1.950; P = 0.1860	NLGF-f (148.4±3.3); NLGF-m (145.5±4.9), Wt-f: (141.0±9.6); Wt-m (155.5±9.9)
Caudate putamen	Genotype: F = 0.06627; P = 0.8009<br>Sex: F = 1.143; P = 0.3044<br>Genotype*sex: F = 2.625; P = 0.1292	NLGF-f (178.9± 3.2); NLGF-m (174.8± 6.5) Wt-f (168.8± 11.3); Wt-m (188.7± 11.7)
Frontal cortex	Genotype: F = 0.04587; P = 0.8337<br>Sex: F = 0.3638; P = 0.5568<br>Genotype*sex: F = 0.9255; P = 0.3536	NLGF-f (154.4± 4.1); NLGF-m (151.8± 5.9) Wt-f (149.0± 11.4); Wt-m (160.3± 10.1)
Hippocampus	Genotype: F = 0.01920; P = 0.8919<br>Sex: F = 1.264; P = 0.2812<br>Genotype*sex: F = 2.417; P = 0.1440	NLGF-f (166.0±3.3); NLGF-m (163.1±6.0) Wt-f (154.4±10.3); Wt-m (172.7±10.3)
Parietal-temporal cortex	Genotype: F = 0.07826; P = 0.7841<br>Sex: F = 0.3469; P = 0.5660<br>Genotype*sex: F = 0.9059; P = 0.3586	NLGF-f (136.6± 3.5); NLGF-m (134.4± 4.5) Wt-f (132.5± 10.1); Wt-m (141.9± 9.0)
Occipital cortex	Genotype: F = 0.0003403; P = 0.9856<br>Sex: F = 1.092; P = 0.3151<br>Genotype*sex: F = 1.090; P = 0.3156	NLGF-f (139.1± 3.0); NLGF-m (139.1± 5.8) Wt-f (132.8± 9.6); Wt-m: (145.7± 8.3)
Medulla	Genotype: F = 0.3093; P = 0.5876<br>Sex: F = 1.762; P = 0.2072<br>Genotype*sex: F = 3.027; P = 0.1055	NLGF-f (123.2± 3.3); NLGF-m (121.2± 3.9) Wt-f: (117.4± 6.5); Wt-m: (132.3± 7.0)
Midbrain	Genotype: F = 0.01107; P = 0.9178<br>Sex: F = 1.318; P = 0.2716<br>Genotype*sex: F = 3.523; *P = 0.0831*	NLGF-f (170.8± 4.0); NLGF-m (165.7± 5.2) Wt-f (156.8± 8.2); Wt-m (178.2± 13.7)
Pons	Genotype: F = 0.05223; P = 0.8228<br>Sex: F = 1.464; P = 0.2479<br>Genotype*sex: F = 3.060; P = 0.1038	NLGF-f (141.8± 3.6); NLGF-m (138.5± 4.6) Wt-f (132.5± 7.7); Wt-m (150.5± 10.7)
Thalamus	Genotype: F = 0.03420; P = 0.8561<br>Sex: F = 1.032; P = 0.3283<br>Genotype*sex: F = 3.192; *P = 0.0973*	NLGF-f (175.0±4.2); NLGF-m (169.3±4.5) Wt-f (160.5±9.0); Wt-m: 181.1±14.8
Cerebellum	Genotype: F = 0.1056; P = 0.7504<br>Sex: F = 2.473; P = 0.1399<br>Genotype*sex: F = 3.498; *P = 0.0841*	NLGF-f (128.5±2.8); NLGF-m (127.0±4.0) Wt-f (121.1±6.7); Wt-m (137.4±7.1)

Abbreviations: AUC: area under the curve; CB1: cannabinoid receptor 1; dF: degrees of freedom; f: female; m: male; NLGF: $App^{NL\text{-}G\text{-}F}$; wt: wild-type.

Table S3: AUC data and statistics (2-way ANOVA with Šídák's multiple comparisons test) of the 12mo [^{18}F]MAGL-2102 scans

**Region**	**Two-way ANOVA** (dF: 1,12)	**Holm-Šídák post-hoc, (AUC mean±SEM)**
Whole brain	Genotype: F = 0.09491; P = 0.7633<br>Sex: F = 0.07598; P = 0.7875<br>Genotype*sex: F = 0.07598; P = 0.7875	NLGF-f (109.7±6.2); NLGF-m (109.7±2.5)<br>Wt-f (109.8±4.5); Wt-m (112.2±2.6)
Caudate putamen	Genotype: F = 0.2247; P = 0.6440<br>Sex: F = 0.01404; P = 0.9076<br>Genotype*sex: F = 0.07231; P = 0.7926	NLGF-f (140.6±7.4); NLGF-m (138.5±3.0)<br>Wt-f (141.7±6.6); Wt-m (142.5±3.6)
Frontal cortex	Genotype: F = 0.07231; P = 0.7926<br>Sex: F = 0.000; P > 0.9999<br>Genotype*sex: F = 0.7387; P = 0.4069	NLGF-f (140.6±8.9); NLGF-m (135.2±5.7)<br>Wt-f (140.1±5.5); Wt-m (145.5±3.4)
Hippocampus	Genotype: F = 0.001075; P = 0.9744<br>Sex: F = 0.03005; P = 0.8653<br>Genotype*sex: F = 0.01484; P = 0.9051	NLGF-f (118.9±7.8); NLGF-m (119.2±4.0)<br>Wt-f (118.1±5.3); Wt-m (119.7±2.9)
Parietal-temporal cortex	Genotype: F = 0.03125; P = 0.8626<br>Sex: F = 0.1441; P = 0.7109<br>Genotype*sex: F = 0.2095; P = 0.6553	NLGF-f (114.4±7.9); NLGF-m (114.0±4.5)<br>Wt-f (112.8±5.3); Wt-m (117.4±2.7)
Occipital cortex	Genotype: F = 0.1053; P = 0.7511<br>Sex: F = 1.139; P = 0.3068<br>Genotype*sex: F = 0.000396; P = 0.9845	NLGF-f (95.1±7.4); NLGF-m (100.6±4.4)<br>Wt-f (93.5±4.7); Wt-m (98.9±2.7)
Medulla	Genotype: F = 0.2239; P = 0.6445<br>Sex: F = 1.660; P = 0.2219<br>Genotype*sex: F = 1.024; P = 0.3314	NLGF-f (65.1±1.4); NLGF-m (70.3±0.9)<br>Wt-f (66.3±2.7); Wt-m (67.0±3.1)
Midbrain	Genotype: F = 0.1085; P = 0.7476<br>Sex: F = 0.1745; P = 0.6835<br>Genotype*sex: F = 0.1393; P = 0.7155	NLGF-f (90.8±4.3); NLGF-m (93.5±1.6)<br>Wt-f (93.2±4.2); Wt-m (93.3±2.8)
Pons	Genotype: F = 0.1196; P = 0.7354<br>Sex: F = 0.3446; P = 0.5680<br>Genotype*sex: F = 1.069; P = 0.3215	NLGF-f (69.5±2.3); NLGF-m (74.1±0.9)<br>Wt-f (71.4±3.9); Wt-m (70.2±3.3)
Thalamus	Genotype: F = 0.2735; P = 0.6105<br>Sex: F = 0.005105; P = 0.9442<br>Genotype*sex: F = 0.008895; P = 0.9264	NLGF-f (113.1±6.2); NLGF-m (112.4±2.7)<br>Wt-f (115.0±4.8); Wt-m (115.1±2.8)
Cerebellum	Genotype: F = 0.08612; P = 0.7742<br>Sex: F = 3.495; *P = 0.0862*<br>Genotype*sex: F = 0.4214; P = 0.5285	NLGF-f (74.7±2.8); NLGF-m (81.4±1.5)<br>Wt-f (77.2±2.9); Wt-m (80.5±3.3)

Abbreviations: AUC: area under the curve; dF: degrees of freedom; f: female; m: male; MAGL: monoacylglycerol lipase; NLGF: *App*$^{NL\text{-}G\text{-}F}$; wt: wild-type.

Supplementary figures

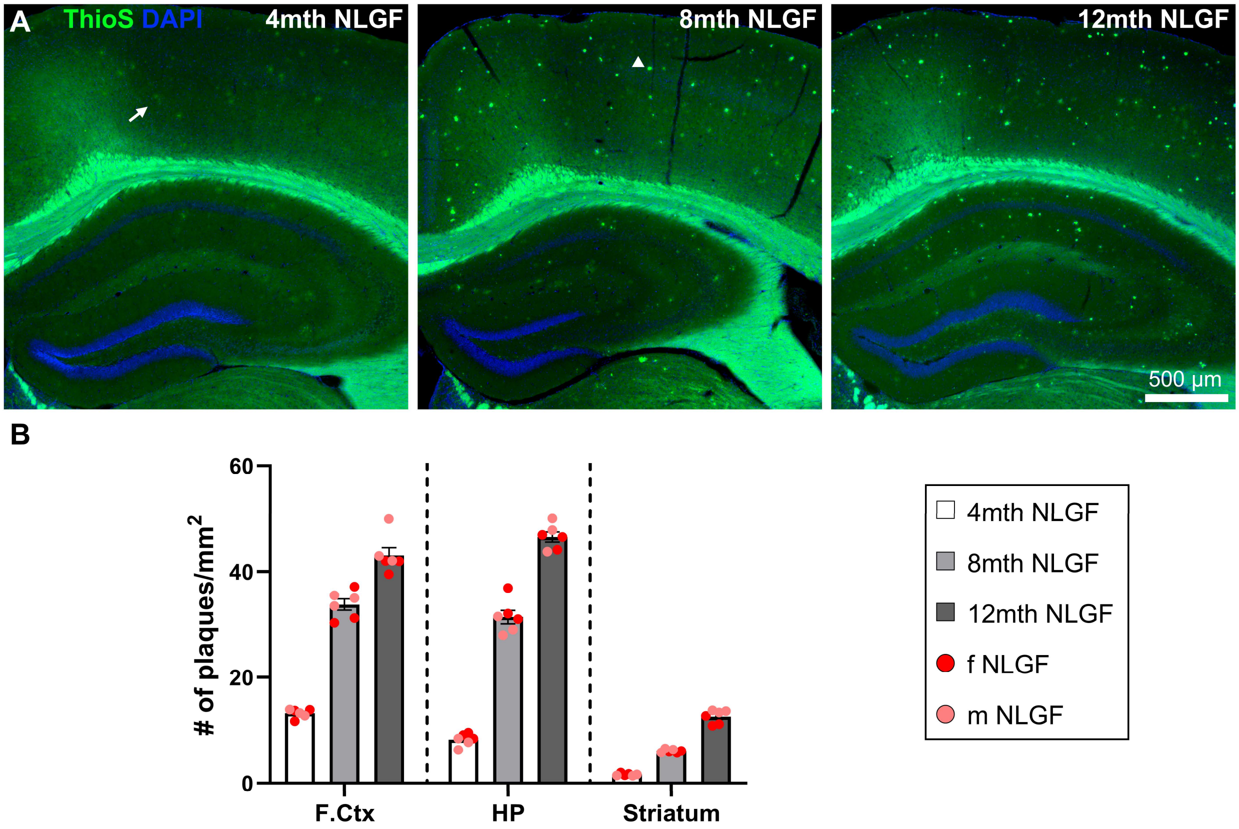

Figure S1: β-Amyloid staging in *App*$^{NL\text{-}G\text{-}F}$ mice at 4-, 8- and 12-months of age. **A)** Representative images of thioflavin-S (green) labelling of β-amyloid plaques, and DAPI (blue) at each age in *App*$^{NL\text{-}G\text{-}F}$ (NLGF) mice. Images demonstrate the increase of plaque over age in the cortex and hippocampus. Diffuse plaques (white arrow) are frequent at 4-months, and cored plaques (white arrowhead) are rare. Plaques greatly increase at 8- and then 12-months, including a high accumulation of dense-cored plaques. **B)** We quantified total plaques (diffuse and cored) in frontal cortex (F.Ctx), hippocampus (HP) and striatum at the three ages (n = 3/sex/age). Frontal cortex exhibits the greatest regional plaque load at 4-months which increases as a function of age (Y = 3.733*X+0.1707). The hippocampus increases at a higher rate (Y = 4.779*X-9.482) and has more plaque (per area) than the cortex at 12-months. Striatal (and caudate putamen; Y = 1.366*X-4.146)

plaque is the lowest of the assessed regions at all 3 ages. Notably, *App*$^{NL\text{-}G\text{-}F}$ mice at 4-months of age represent an early-stage β-amyloid plaque pathology, with a majority diffuse plaques and minimal pathology in the hippocampus (Thal phase 1-2). The *App*$^{NL\text{-}G\text{-}F}$ mid-stage at 8-months exhibits significantly higher amyloid load, yet the hippocampus is still not as robustly affected as the cortex (Thal phase 2-3). Finally, 12-months represents late-stage β-amyloid plaque as demonstrated by robust accumulation in the hippocampus and cortex, with the striatum additionally impacted to a similar degree as the cortex is at the early-stage (Thal phase 3-5; [19]). We observed subtle sex differences in plaque: 4-month frontal cortical plaque trends to an increase in female vs male *App*$^{NL\text{-}G\text{-}F}$ ($P = 0.0913$) and 12-month striatal plaque is significantly higher in male *App*$^{NL\text{-}G\text{-}F}$ (***P = 0.0265***).

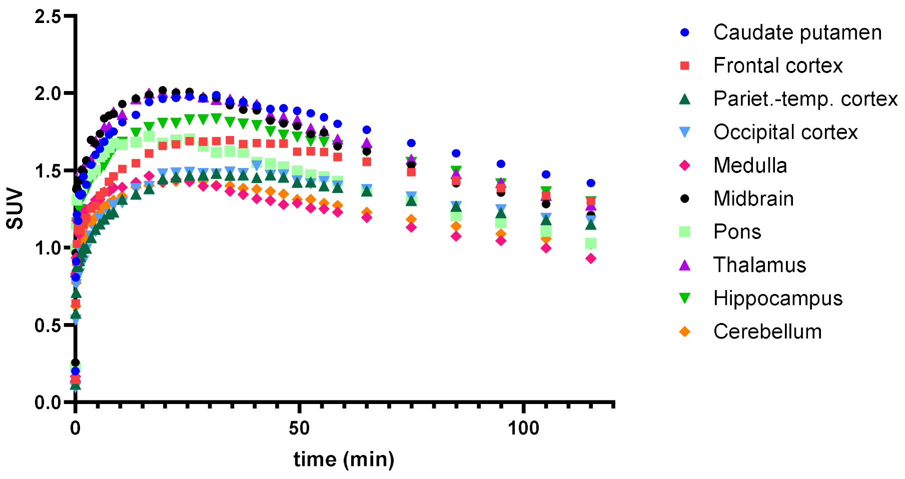

Figure S2: Representative TACs of [^{18}F]FMPEP-d_2 uptake in various brain regions (12mo-f wt mice, mean; n = 4).

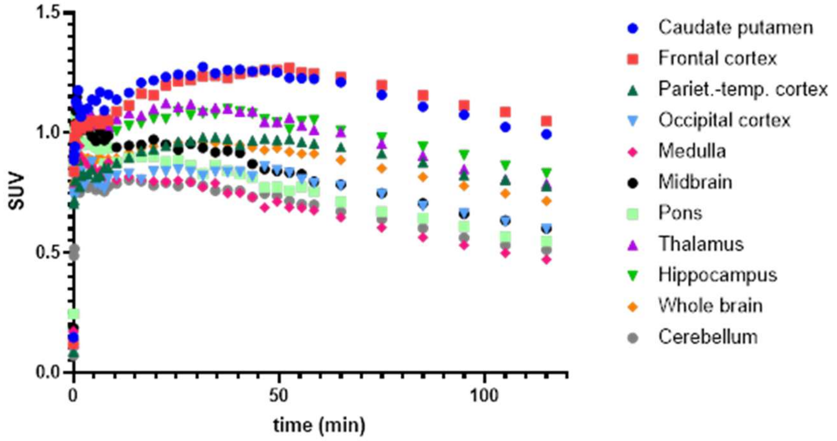

Figure S3: Representative TACs of [^{18}F]MAGL-2102 uptake in various brain regions (4mo-f wt mice, mean; n = 4).

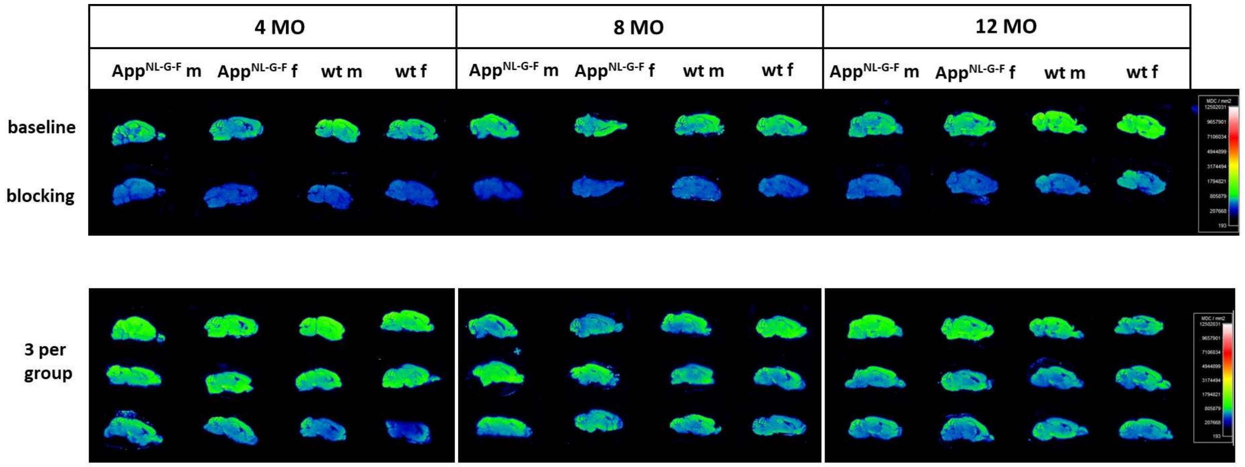

Figure S4: Autoradiography of sagittal brain sections with [^{18}F]FMPEP-d_2 under baseline and blocking (10 µM unlabelled FMPEP-d_2) conditions (upper part) as well as 3 per group under baseline conditions for inter-group comparisons.

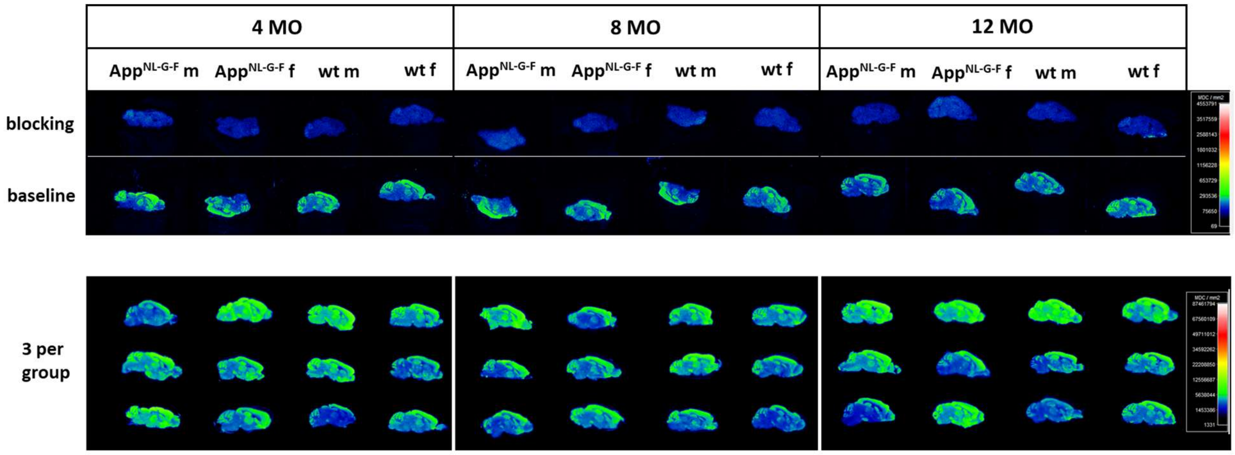

Figure S5: Autoradiography of sagittal brain sections with [^{18}F]MAGL-2102 under baseline and blocking (10 µM unlabelled MAGL-2102) conditions (upper part) as well as 3 per group under baseline conditions for inter-group comparisons.

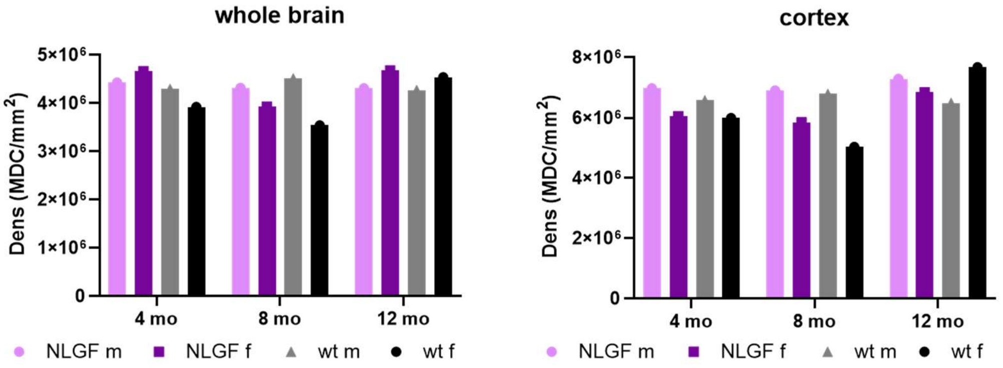

Figure S6: Quantification of the autoradiography signal in whole brain and cortex; average signal density±SEM of 3 sagittal sections per group (shown in Supplemental Figure 5).

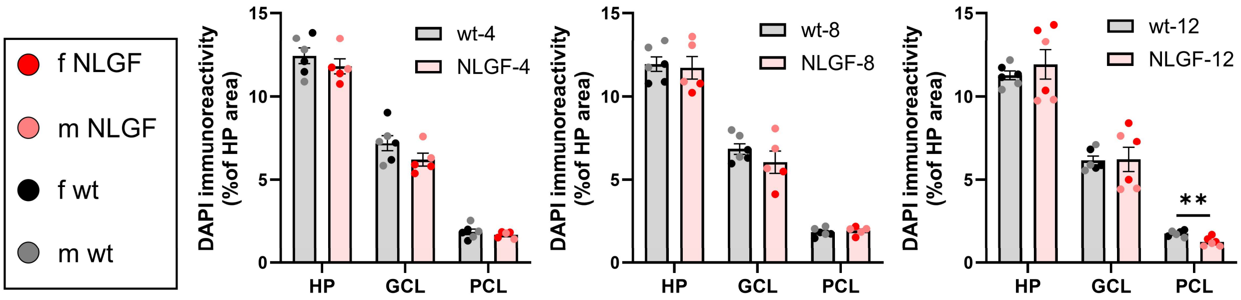

Figure S7: Supplementary data to **Figure 3**. We quantified DAPI immunoreactivity in the total hippocampus (HP), granular cell layer (GCL) and combined CA1 and CA3 pyramidal cell layers (PCL) at 4-, 8- and 12-months of age in wild-type (wt) and *App*$^{NL\text{-}G\text{-}F}$ (NLGF) mice. No significant genotype differences were detected at 4- (P = 0.5998, P = 0.3654, P = 0.5998, respectively) or 8-months (P = 0.8173, P = 0.6416, P = 0.8173, respectively). At 12-months, a significant loss of DAPI was detected in NLGF PCL (***P = 0.0094***), but not in HP (P = 0.7436) or GCL (P = 0.9462). Data are mean +/- SEM; multiple unpaired t-tests with Holm-Šídák correction (n = 5-6/genotype/age). **P < 0.01.

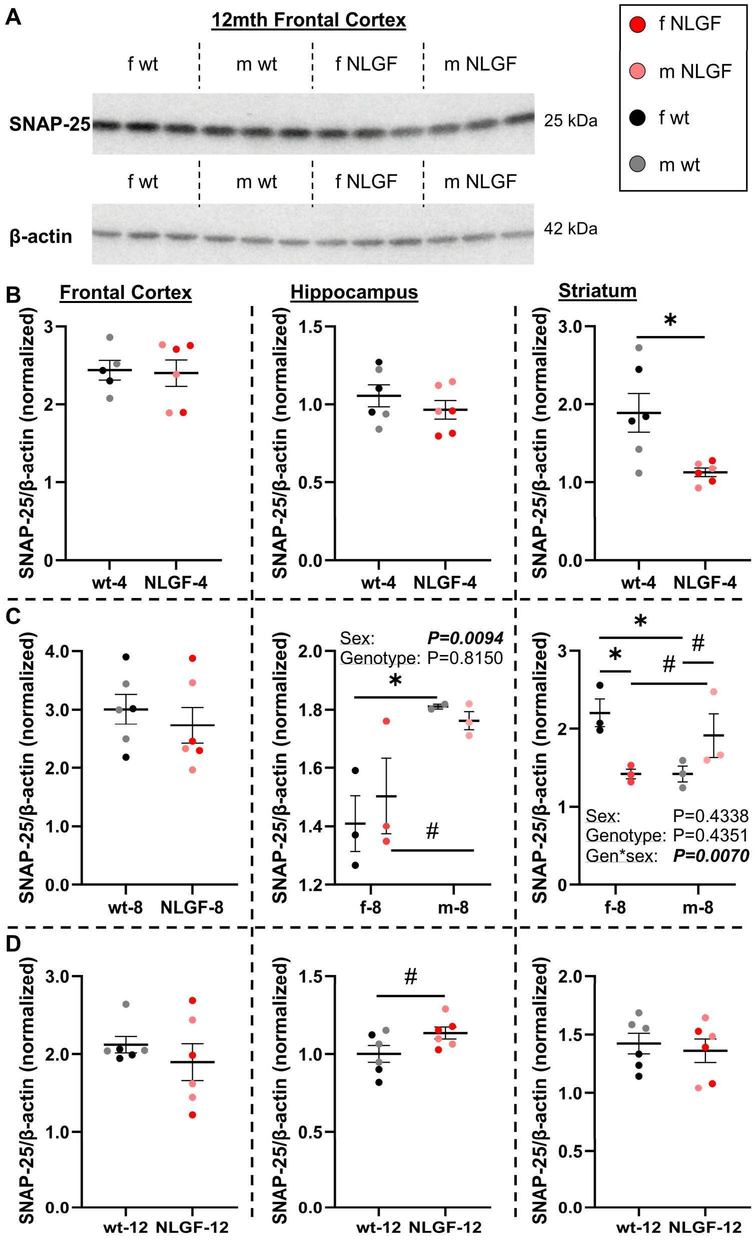

Figure S8: Protein levels of SNAP-25 were assessed by western blot at 4-, 8-, and 12-months of age in wt control and *App*$^{NL\text{-}G\text{-}F}$ mice (*n* = 5-6/genotype/age). **A)** Representative blot of SNAP-25 and β-actin (loading control). This SNAP-25 is on the same gel shown in **Figure 4A** for CB1, normalized to the same β-actin bands which are shown again here. SNAP-25 expression was analyzed and normalized to β-actin at each age in the frontal cortex, hippocampus and striatum homogenates. **B**) At 4-months, no significant differences were detected in the frontal cortex (P =

0.8701) or hippocampus (P = 0.3564), yet early-stage *App*$^{NL\text{-}G\text{-}F}$ mice exhibit a loss of striatal SNAP-25 compared to wt (***P = 0.0129***). **C)** No significant differences were detected in the 8-month frontal cortex (P = 0.5050). Similarly, there was no genotype differences in 8-month hippocampus (P = 0.8150, F(1,7) = 0.0590), though there was a significant sex effect (***P = 0.0094***, F(1,7) = 12.55) with significantly less SNAP-25 in wt females compared to males (***P = 0.0461***) and a trend within *App*$^{NL\text{-}G\text{-}F}$ mice (*P = 0.0759*; two-way ANOVA, Holm-Šídák post-hoc). In the 8-month striatum, no overall genotype (P = 0.4351, F(1,8) = 0.6750) or sex (P = 0.4338, F(1,8) = 0.6789) differences were detected; however, the genotype*sex interaction effect was significant (***P = 0.0070***, F(1,8) = 12.95), with lower striatal SNAP-25 levels in female *App*$^{NL\text{-}G\text{-}F}$ mice compared to wt female (***P = 0.0280***) and in wt male compared to wt female (***P = 0.0279***, two-way ANOVA, Holm-Šídák post-hoc). Male 8-month *App*$^{NL\text{-}G\text{-}F}$ mice trended to higher striatal SNAP-25 compared to female transgenics (*P = 0.0853*) and male controls (*P = 0.0851*). **D)** Finally, in the 12-month mice, no significant differences were detected in the frontal cortex (P = 0.4067), a trend to more SNAP-25 protein levels in the *App*$^{NL\text{-}G\text{-}F}$ hippocampus compared to wt controls (*P = 0.0717*), and no significant genotype differences in the striatum (P = 0.6540). Normalized protein values are relative to exposure time and should not be compared between gels and graphs. Within each region, 4- and 12-month mice were on the same gels; see **Figure S12** for age comparisons. Data are mean+/-SEM; unpaired t-test or two-way ANOVA with Holm-Šídák post-hoc. #P < 0.10; *P < 0.05. CB1: Cannabinoid Receptor 1; kDa: kilodalton; NLGF: *App*$^{NL\text{-}G\text{-}F}$; SNAP-25: Synaptosomal-Associated Protein, 25 kDa; wt: wild-type.

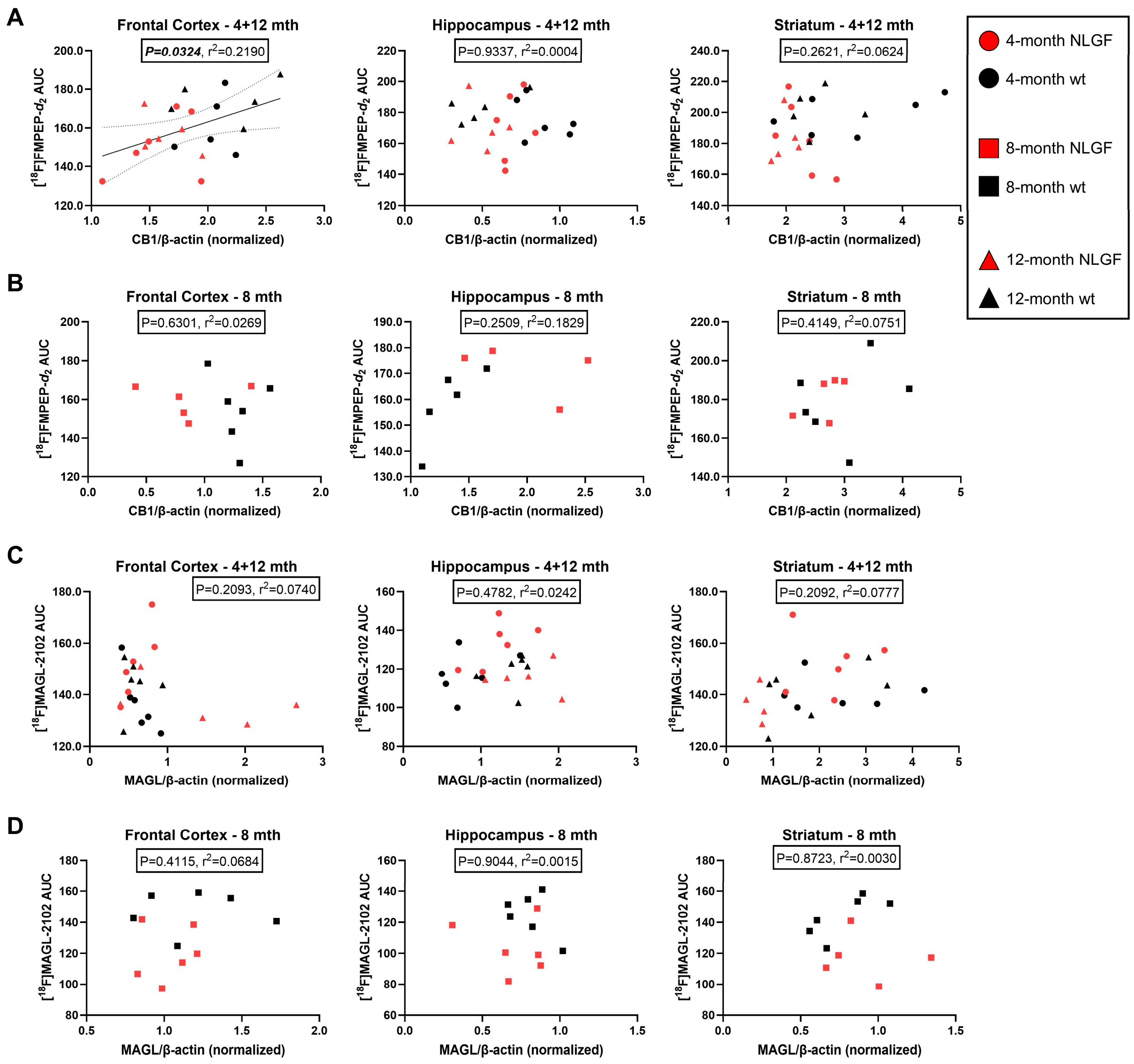

Figure S9: CB1 protein levels were correlated with [^{18}F]FMPEP-d_2 AUC data in the grouped 4- and 12-month mice (**A**) and in 8-month mice separately (**B**), split by frontal cortex, hippocampus and striatum. A significant positive correlation was detected between CB1 protein and PET availability in the frontal cortex of the 4- and 12-month mice (***P = 0.0324***, r^2 = 0.2190); linear regression line (+/- 95% confidence intervals) was plotted (Y = 19.43*X + 124.3). No other correlations were detected for CB1 (**A, B**), or for any region in the 4- and 12-month (**C**) or 8-month (**D**) MAGL protein vs. [^{18}F]MAGL-2102 AUC correlations. Normalized protein values (Y axes)

are relative to exposure time and should not be compared between gels and graphs. Within each region, 4- and 12-month mice were on the same gels, and 8-month mice were separate. AUC: area under the curve; CB1: Cannabinoid Receptor 1; MAGL: monoacylglycerol lipase; NLGF: App^{NL-G-F}; PET: positron emission tomography; wt: wild-type.

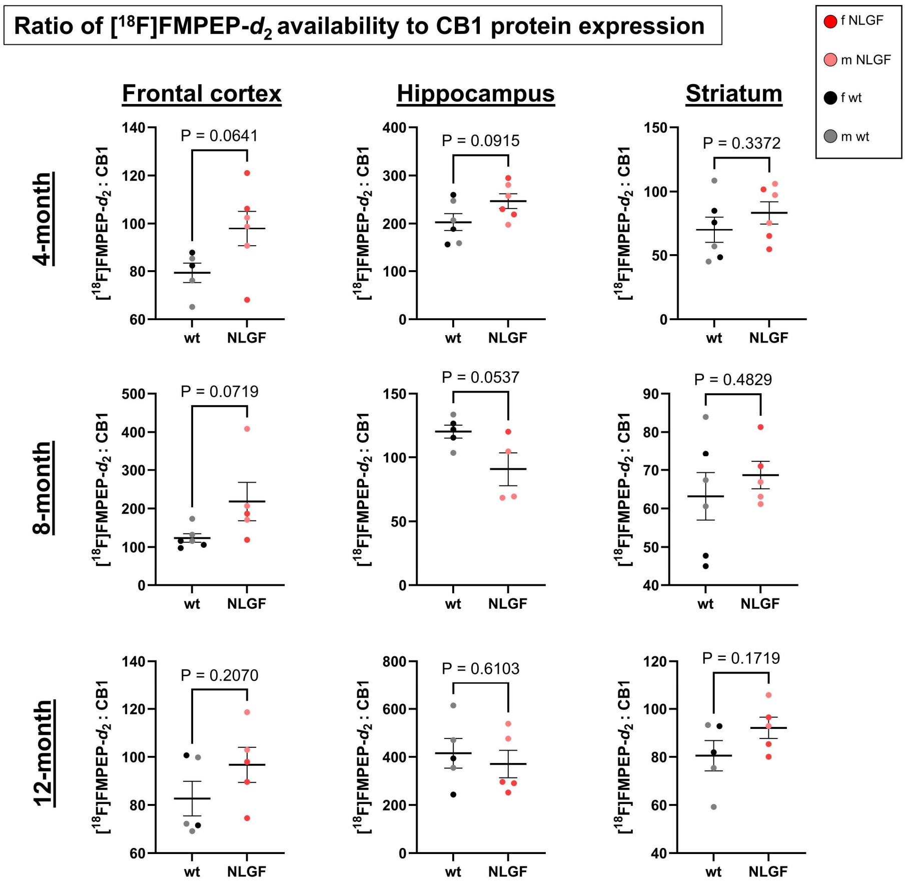

Figure S10: The ratio of [^{18}F]FMPEP-d_2 availability to CB1 protein levels was calculated and graphed for each age and region assessed. Trends to greater [^{18}F]FMPEP-d_2 availability per CB1 protein expressed was determined in App^{NL-G-F} mice at 4-months in the frontal cortex and hippocampus, and at 8-months in the frontal cortex. Conversely, 8-month App^{NL-G-F} mice trended to less availability per protein levels in the hippocampus. All other analyses were non-significant.

Normalized protein values are relative to exposure time and should not be compared between gels and graphs. Analysis was with unpaired t-test and the P values are reported in each graph. Data are mean+/-SEM. CB1: Cannabinoid Receptor 1; NLGF: *App*$^{NL\text{-}G\text{-}F}$; PET: positron emission tomography; wt: wild-type.

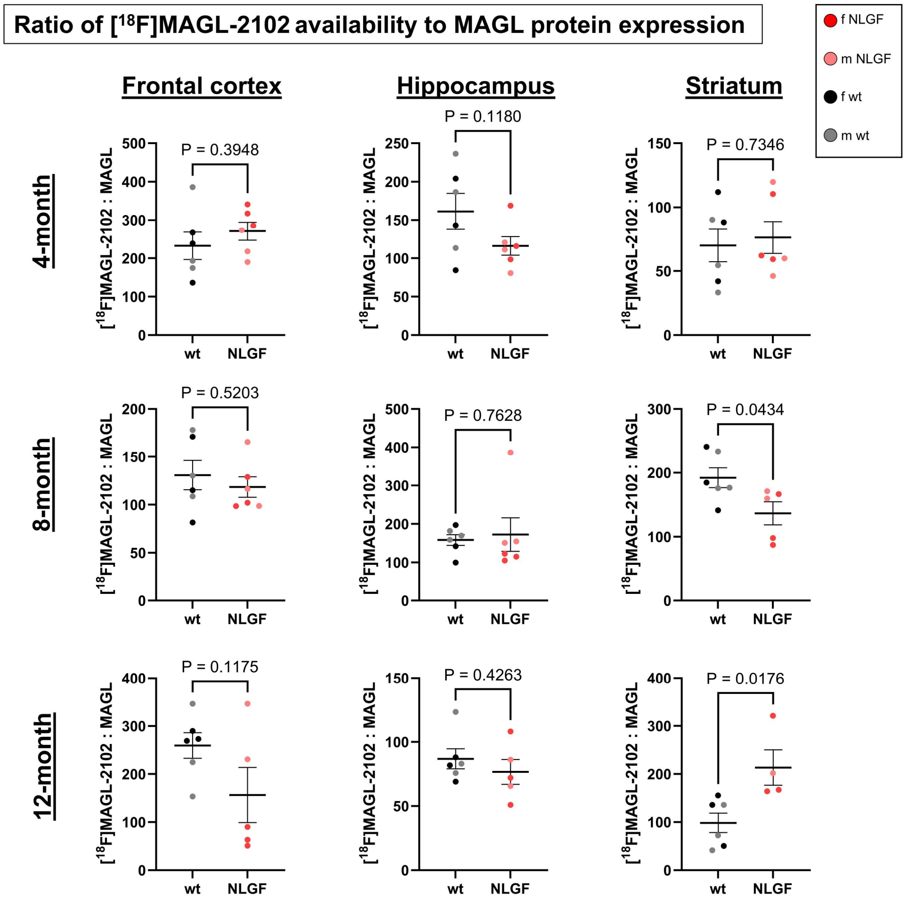

Figure S11: The ratio of [^{18}F]MAGL-2102 availability to MAGL protein levels was calculated and graphed for each age and region assessed. Significantly lower MAGL availability per protein expressed was detected in the 8-month *App*$^{NL\text{-}G\text{-}F}$ striatum, and significantly greater availability per protein in the 12-month *App*$^{NL\text{-}G\text{-}F}$ striatum. All other analyses were non-significant. Normalized protein values are relative to exposure time and should not be compared between gels and graphs.

Analysis was with unpaired t-test and the P values are reported in each graph. Data are mean+/- SEM. MAGL: monoacylglycerol lipase; NLGF: $App^{NL\text{-}G\text{-}F}$; PET: positron emission tomography; wt: wild-type.

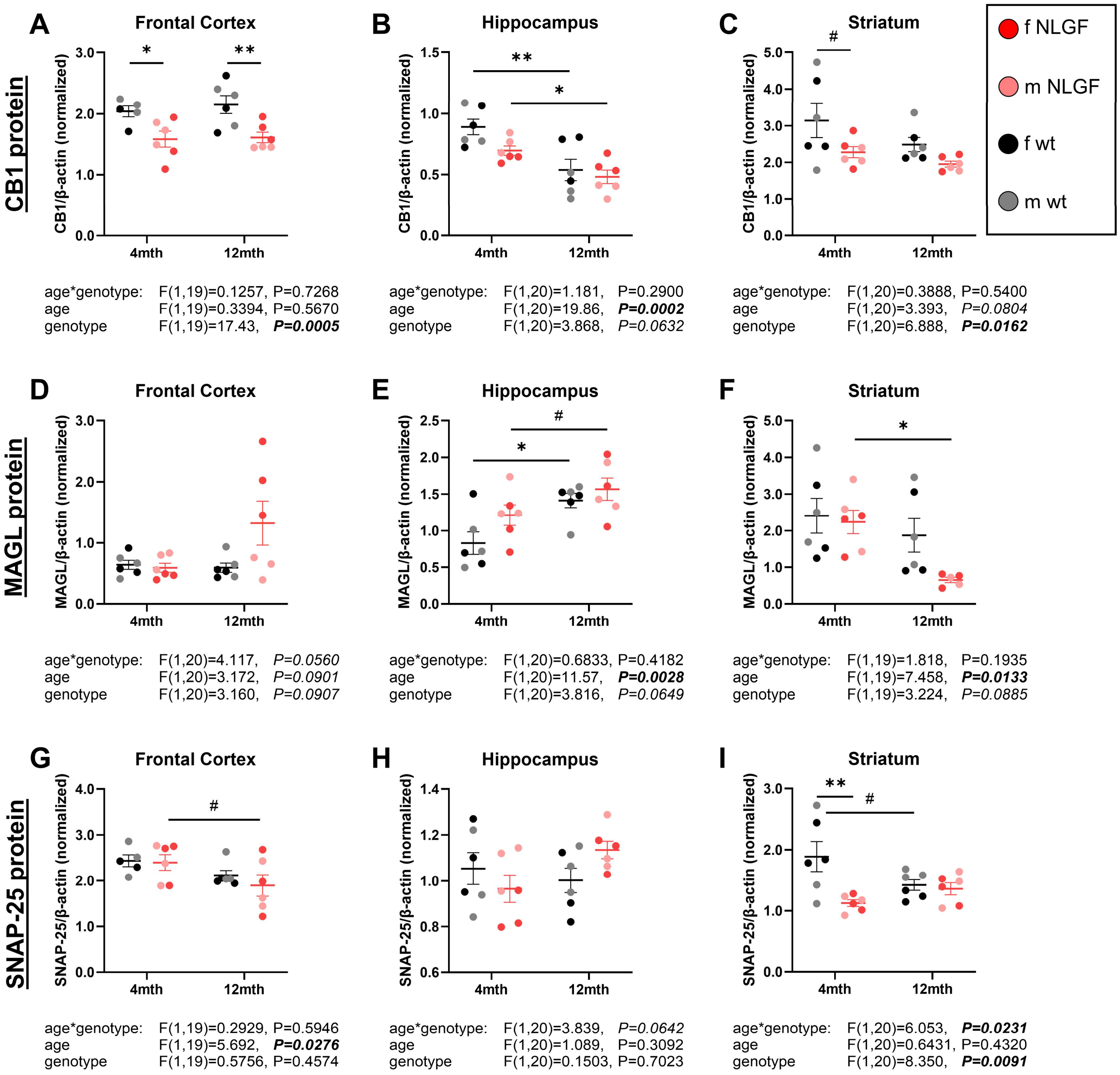

Figure S12: CB1, MAGL and SNAP-25 protein levels were assessed for age effects between 4- and 12-months in wt and $App^{NL\text{-}G\text{-}F}$ mice, complementing the full western dataset (see **Figures 4,**

5 and S8, respectively). 4- and 12-month samples were assessed on the same gels for westerns within each region, and henceforth we were able to assess age effects, albeit with the 8-month timepoint omitted. Analyses were conducted as a two-way ANOVA (reported in figure) with Holm-Šídák correction for multiple comparisons (reported in legend text). **A**) Frontal cortical CB1 protein did not change by age, but had a significant genotype effect with lower levels in $App^{NL\text{-}G\text{-}F}$ vs. wt mice at 4- (***P = 0.0162***) and 12-months of age (***P = 0.0078***). **B**) Hippocampal CB1 protein decreased by age and trended to a decrease by $App^{NL\text{-}G\text{-}F}$ genotype; significantly lower in 12-month wt (***P = 0.0017***) and $App^{NL\text{-}G\text{-}F}$ mice (***P = 0.0272***) vs. at 4-months. **C**) Striatal CB1 protein was significantly lower overall in $App^{NL\text{-}G\text{-}F}$ mice and trended to an age-related decrease, with multiple comparisons determining a trend to lower $App^{NL\text{-}G\text{-}F}$ protein at 4-months (*P = 0.0641*), but not at 12-months (P = 0.1725), compared to wt. **D**) Frontal cortical MAGL protein trended to age, genotype and age*genotype effects. **E**) Hippocampal MAGL protein increased over age in wt (***P = 0.0144***) and $App^{NL\text{-}G\text{-}F}$ (*P = 0.0836*) mice, with a trend to more in the $App^{NL\text{-}G\text{-}F}$ (vs. wt) genotype effect. **F**) Striatal MAGL protein decreased with age specifically in the 12-month $App^{NL\text{-}G\text{-}F}$ mice (***P = 0.0218***) and not in the wt mice (P = 0.3290); genotype effects were trending. **G**) Frontal cortical SNAP-25 protein was significantly reduced with age overall, more so in $App^{NL\text{-}G\text{-}F}$ (*P = 0.0924*) than wt (P = 0.2179) mice. **H**) A trend to an age*genotype interaction effect was observed in hippocampal SNAP-25 protein levels. **I**) Significant genotype and age*genotype effects were observed in striatal SNAP-25 protein, with a significant deficit in 4-month (***P = 0.0023***) but not 12-month (P = 0.7646) $App^{NL\text{-}G\text{-}F}$ mice, and a trend to a loss over age in wt (*P = 0.0628*) but not in $App^{NL\text{-}G\text{-}F}$ (P = 0.2547) mice. Data are mean+/-SEM. #P < 0.10; *P < 0.05; **P < 0.05. CB1: Cannabinoid Receptor 1; MAGL: monoacylglycerol lipase; NLGF: $App^{NL\text{-}G\text{-}F}$; SNAP-25: Synaptosomal-Associated Protein, 25 kDa; wt: wild-type.

Frontal Cortex

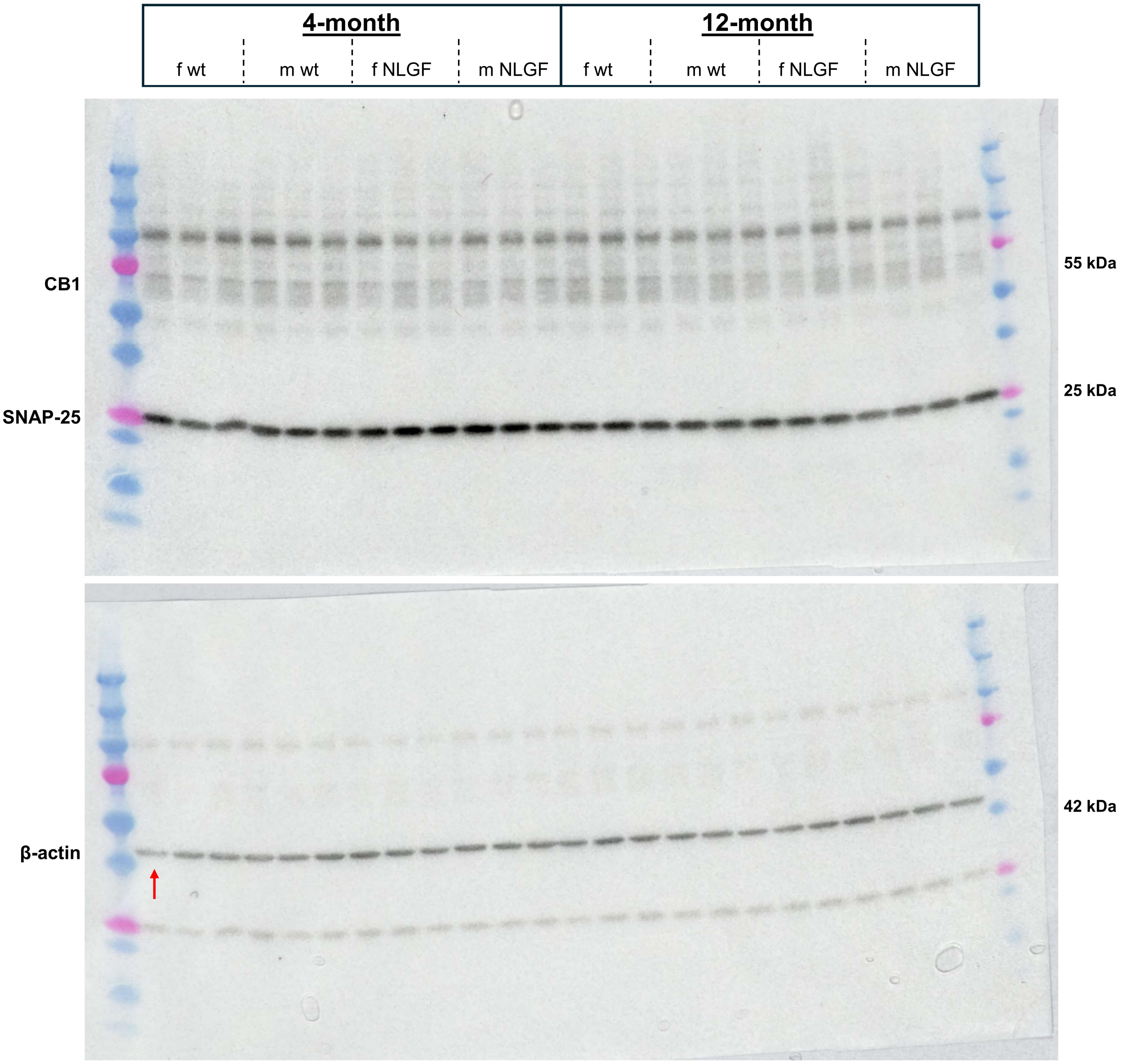

Figure S13: Full western blot images for CB1 (**Figure 4**) and SNAP-25 (**Figure S8**) in the frontal cortex of 4- and 12-month wt and *App*$^{NL\text{-}G\text{-}F}$ mice (NLGF). One 4-month wt mouse (first lane, red arrow) was excluded due to low β-actin signal.

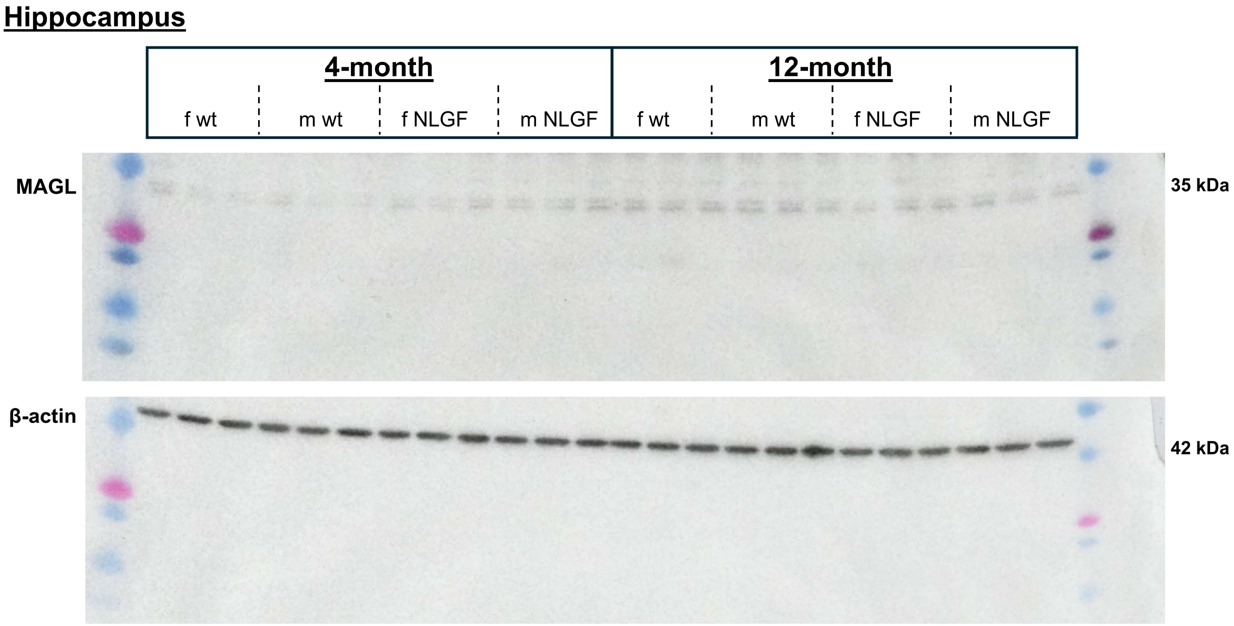

Figure S14: Western blot images for MAGL (**Figure 5**) in the hippocampus of 4- and 12-month wt and *App*$^{NL\text{-}G\text{-}F}$ mice (NLGF). Images were cropped above 50 kDA because another target was tested on these gels, but not included in the study. This target was overexposed at the exposure time needed to develop the MAGL signal.
